# Redevelopment of mental health first aid guidelines for substance use problems: a Delphi study

**DOI:** 10.1186/s40359-024-01561-8

**Published:** 2024-02-13

**Authors:** Judith Wright, Kathryn J. Chalmers, Alyssia Rossetto, Nicola J. Reavley, Claire M. Kelly, Anthony F. Jorm

**Affiliations:** 1 Centre for Mental Health and Community Wellbeing, Melbourne School of Population and Global Health , The University of Melbourne, 3010 Parkville, VIC Australia; 2 Mental Health First Aid Australia, Level 18, 150 Lonsdale Street, 3000 Melbourne, VIC Australia

**Keywords:** Mental health first aid, Substance use, Helping behaviour, Delphi method, Expert consensus, Community guidelines

## Abstract

**Background:**

Substance use problems have a major impact on the physical and mental health of individuals, families and communities. Early intervention may have a positive effect on recovery and treatment outcomes for those with substance use problems, reducing related risk and harm. Separate mental health first aid guidelines on how a member of the public could assist someone experiencing or developing alcohol use and drug use problems in high income Western countries were developed using Delphi expert consensus in 2009 and 2011, respectively. This study aimed to synthesise and update these two original guidelines to reflect current evidence and best practice.

**Methods:**

The Delphi expert consensus method was used to determine the inclusion of statements in the redeveloped guidelines. A questionnaire was developed using previously endorsed helping statements from the original guidelines on alcohol and drug use problems, as well as relevant content identified in systematic searches of academic and grey literature. Three panels of experts (people with lived experience, support people and professionals) rated statements over three consecutive online survey rounds to determine the importance of their inclusion in the guidelines. Statements endorsed by at least 80% of each panel were included.

**Results:**

103 panellists completed all three survey rounds. They rated 469 statements and endorsed 300 of these for inclusion in the redeveloped guidelines.

**Conclusions:**

This study has developed a broader and more comprehensive set of guidelines for how to support a person experiencing or developing a substance use problem. The redeveloped guidelines provide more detail on knowledge about and recognition of substance use problems, approaching and assisting people who want to change or are not ready to change, harm reduction, community-based supports and professional help, but have less on physical first aid actions. Mental Health First Aid International will use these guidelines in future updates of their training courses.

**Supplementary Information:**

The online version contains supplementary material available at 10.1186/s40359-024-01561-8.

## Background

Substance use is one of the top ten leading contributors to burden of disease and is associated with a wide range of adverse health, social and economic consequences, including disability and premature mortality [1–4]. Substance use disorders and other harmful patterns of substance use, including binge drinking and illicit drug use, substantially increase the risk of other mental disorders (e.g., depression, drug-induced psychosis), chronic health conditions (e.g.,cancer, cirrhosis), injury due to road-traffic accidents or violence, and death (e.g., overdose, suicide) [1, 5]. Worldwide, the prevalence of substance use problems that would benefit from treatment and support is high. Findings from countries participating in the WHO’s World Mental Health Surveys found the mean lifetime prevalence of alcohol use disorders in all countries combined were 8.6%, ranging from 0.7% in Iraq to 22.7% in Australia, and 3.5% for drug use disorders [6, 7]. Early intervention efforts to ensure prompt engagement with support are needed to reduce substance-related harm and premature mortality.

Treatment and support options such as mutual support, medical and pharmacological treatment, and cognitive-behavioural therapy are effective in reducing the prevalence and harms of substance use problems [8–10]. However, many people living with substance use problems do not seek help or typically only do so after several aspects of their lives are impacted (e.g., health, relationships and finances) [11, 12]. Compared to anxiety and mood disorders, substance use disorders have the lowest probability of treatment contact in the first year of disorder onset and the longest delay from onset to first treatment contact [13]. For those who have sought help for substance use problems, the influence of concerned others such as family, friends, and colleagues has been consistently identified as playing an important role in supporting and motivating a person with substance use problems to initiate treatment and change their behaviour [14–18]. However, despite concerned others being well placed to support the process of early intervention for people with substance use problems, evidence points to the need for significant improvements in their knowledge and skills for providing help [19, 20].

The Mental Health First Aid (MHFA) training program, developed by Kitchener and Jorm in response to low levels of mental health-related knowledge and skills in the general community, teaches members of the public how to assist someone developing a mental health problem, experiencing a worsening of an existing mental health problem or in a mental health crisis. The first aid is given until appropriate professional help is received, or the crisis resolves [21]. Originally developed in Australia in 2000, this program is now disseminated in over 24 countries. A recent systematic review and meta-analysis of 18 controlled trials demonstrated its effectiveness up to six months after completion of training with improvements in participants’ knowledge of mental health conditions, mental health first aid skills, and confidence to help a person with a mental health problem [22]. The content of the program is informed by guidelines developed using the Delphi expert consensus method. This method is widely used in mental health research as a systematic way of incorporating practice-based evidence from experts when experimental and epidemiological methods cannot be used [23]. This method was used to redevelop the original mental health first aid guidelines for a suite of mental health problems and crises including depression, panic attacks, a potentially traumatic event, and psychosis [24–27]. This process of revising the original guidelines has proven valuable in generating more specific guidance for providing mental health first aid and ensuring that the content of the training is up to date and reflects current evidence and best practice.

Guidelines for how concerned others can provide initial support to people experiencing alcohol and drug use problems were developed separately in 2009 and 2011 respectively [28, 29]. WHO’s *Global status report on alcohol and health* recommends that public health policies, strategies and interventions should collectively target alcohol and other psychoactive drug use as their frequent combined use is shown to lead to preventable mortality [7]. As such, this study aimed to use the Delphi expert consensus method to update the previous guidelines for alcohol and drug use problems and combine them into one set of guidelines addressing substance use problems.

## Method

### Delphi method

This redevelopment involved five stages: (1) Literature search, (2) Questionnaire development, (3) Panel recruitment and formation, (4) Delphi survey rounds and data analysis, and (5) Guidelines development. The methodology was informed by past studies that have used the Delphi expert consensus method to redevelop mental health first aid guidelines, for example guidelines for a potentially traumatic event, depression, panic attacks, and psychosis [24–27].

### Literature search

Researchers (JW & KJC) conducted literature searches of peer-reviewed publications, grey literature and books to identify new statements about knowledge and skills that a member of the public may need to offer help to a person experiencing substance use problems. For the purposes of this project, the term “substance use problems” refers to patterns of harmful substance use with the potential to negatively impact a person’s physical and mental health, relationships, employment, finances, and the safety of themselves and others. To retrieve the most accurate results, a search strategy was developed using a combination of the key concepts: ‘substance use problems’, ‘help’, and ‘first aider’. For example, substance use problems terms included ‘alcohol’, ‘drink’, ‘drug’, ‘substance’, ‘cannabis’, ‘ecstasy’, ‘amphetamine’, ‘cocaine’’, help terms included ‘help’, ‘aid’, and ‘guide’ and ‘support’, and first aider terms included ‘family’, ‘friend’, ‘someone’, ‘partner’, and ‘loved one’. The keywords were adapted for each database and search engine. Each database search was conducted from 2009 onwards to ensure only new strategies from publications that were not covered in the original guidelines on alcohol and drug use were included. 

Google and Amazon Books search engines were used to identify relevant grey literature such as websites, guides, factsheets, and books. As Google search content varies by geolocation, search engines for five English-speaking countries in which MHFA training is available were searched: Australia (Google.com.au), Canada (Google.ca), New Zealand (Google.nz), United Kingdom (Google.co.uk) and the United States (Google.com). The top 50 website results from each search were retrieved and deduplicated. The remaining results were screened for new and relevant knowledge and strategies. Where possible, incognito or private modes were used to avoid the influence of search algorithms. Website and book results with links to other potentially relevant websites or resources were also reviewed. Results were excluded if they were newspapers, sources depicting personal accounts of experiences such as blogs, forums or novels, or provided information not explicitly related to mental health first aid knowledge and actions for substance use problems.

Databases PsycINFO and Medline were used to search for relevant peer-reviewed literature. The database searches returned 1234 articles, of which 99 were identified as having relevant information following title/abstract screening. No articles were included following full-text screening as they did not contain new knowledge or strategies that were not previously captured by the Google search and most of their content focused on clinical intervention which was out of scope.

**Fig. 1:**
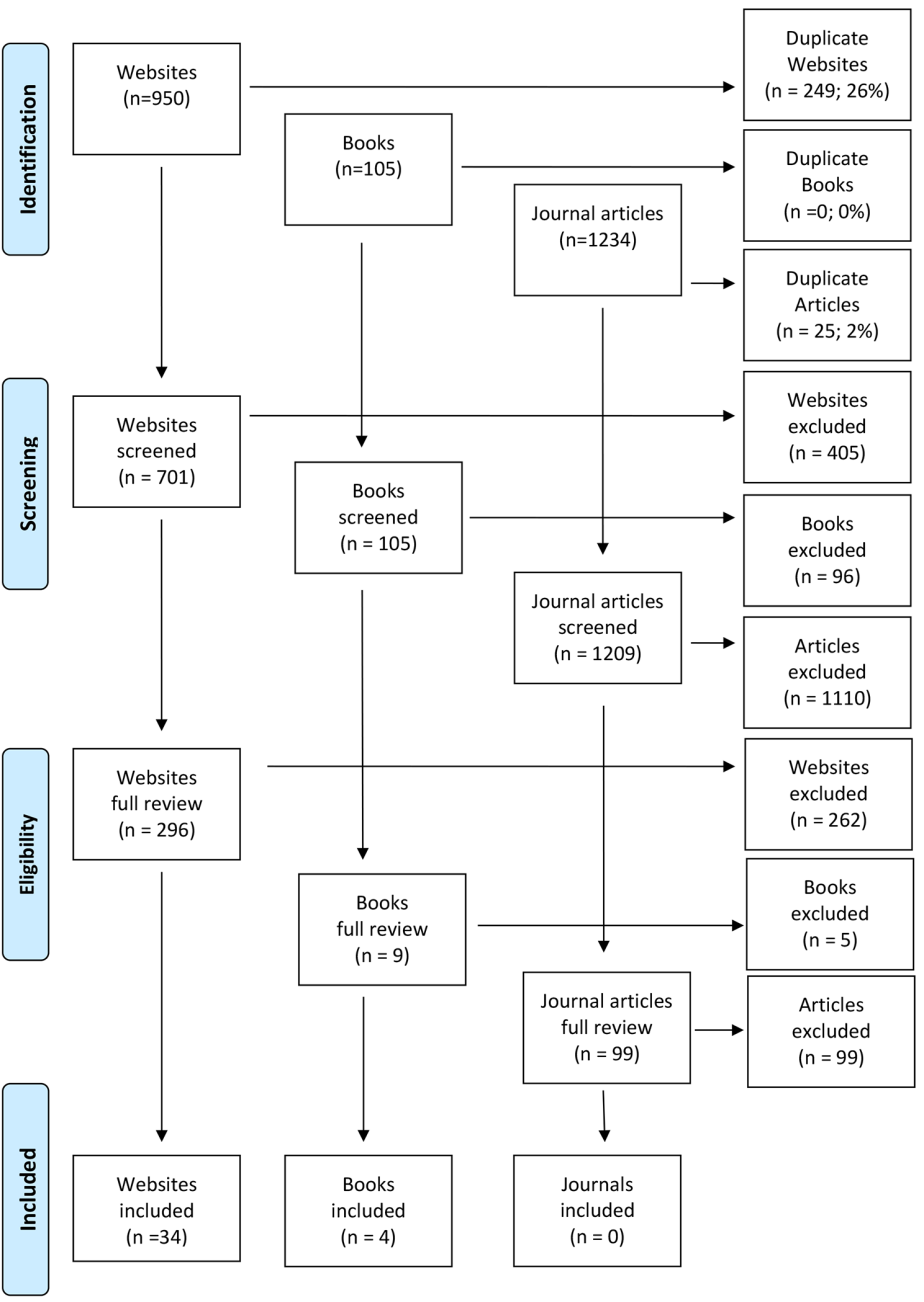
Summary of literature search

The search strategy was deemed comprehensive given the wide range of potentially new first aid strategies retrieved from the literature, as well as the high level of duplication and repetition found across sources. A summary of the literature search is provided in Fig. 1.

### Questionnaire development

The first questionnaire was developed by a working group of researchers (JW, KJC, AR, NJR, AFJ, CMK) in meetings that involved compiling a list of reviewed statements on providing mental health first aid for substance use problems. For statements to be included in the first questionnaire, each statement needed to reflect a single idea describing knowledge required to inform an action or an action on how a first aider could assist a person experiencing a substance use problem. Statements reviewed by the working group included those drafted from new content not reflected in the original guidelines identified in the literature search and endorsed statements from the original guidelines which were revised to: (1) make them applicable to substance use in general, (2) be consistent with the updated definition of mental health first aid, and (3) reduce overlap with other mental health first aid guidelines. For example, helping statements with actions that were congruent across substances were adapted to replace ‘alcohol use’ or ‘drug use’ with ‘substance use’. Statements from the original guidelines were excluded if they no longer aligned with the updated definition of mental health first aid (e.g., provision of physical first aid) or reflected communication skills repeatedly endorsed across previous Delphi studies on developing or redeveloping guidelines for providing mental health first aid for a mental health problem.

All draft statements were reviewed for language, phrasing, and inclusion of examples to ensure their clarity, relevance and actionability. Statements were grouped into themes and categories before the questionnaire was reviewed a final time by the working group to ensure its overall comprehension and to avoid any repetition among statements. Instructions, relevant definitions and a sociodemographic questionnaire for panellists were then added to the survey. In total, the working group revised and approved the inclusion of 385 statements in the first questionnaire.

### Panel recruitment and formation

Panellists were recruited by two researchers (KJC and JW) from Western, high-income countries with comparable health systems. These included Australia, Canada, Europe, New Zealand, the United Kingdom and the United States of America. Panellists were required to speak English, be over 18 years of age, and have expertise relevant to one of the three panels: (1) People with lived experience: People with personal experience of substance use problems, (2) Support people: People who have experience in supporting a person living with substance use problems such as a family member or friend, and (3) Professionals: People with at least 5 years of professional experience working in the field of substance use problems such as health professionals, educators or researchers. People with lived experience and support people also required engagement in activities such as advisory, advocacy or peer support to participate. This was to ensure that their responses reflected broader exposure to people’s experiences of substance use problems, not just the panellists’ experiences alone.

Previous Delphi studies have yielded stable results with at least 20 participants per Delphi panel [23]. This study aimed to recruit a minimum of 30 participants per panel to meet the minimum requirement following attrition across the three survey rounds.

People with lived experience and support people were recruited with support from mental health and/or substance use consumer and support people organisations and groups who agreed to advertise the study amongst their networks and members. Professional experts were recruited from relevant academic journals, professional bodies, alcohol and drug use services and mental health and/or substance use advocacy organisations. Researchers who were identified through their published work in the field of alcohol and drug use were also directly invited to participate. The study was also advertised by Mental Health First Aid Australia to their network of Instructors and through their newsletter, website, and social media. Accredited Mental Health First Aid Instructors often hold multiple roles as people with lived experience, support people and professionals, making them eligible to participate. To limit potential bias due to familiarity with the original guidelines from delivering the Mental Health First Aid courses, no more than 50% of each panel could comprise Mental Health First Aid Instructors.

Individuals who expressed interest to participate were provided with a Plain Language Statement containing more information about the project. Those who agreed to participate and met eligibility criteria were assigned a panel or were given the opportunity to select the panel that best reflected their expertise if they met criteria for more than one panel before they were emailed a link to the Round 1 survey. Participants were offered an honorarium equivalent to $AUD200 for completing all three survey rounds.

### Delphi consensus survey rounds and data analysis

Data were collected over three consecutive survey rounds between February and November 2022 (see Additional file 1 for copies of surveys from each round). Survey rounds were hosted online via Qualtrics. The Round 1 survey included the statements on providing mental health first aid for substance use problems, open text-box questions that prompted panellists to provide comments to improve or offer new suggestions to the survey statements, and sociodemographic questions.

In each survey round, panellists rated a series of statements according to how important they thought their inclusion was in mental health first aid guidelines for substance use problems using a 5-point Likert scale (‘essential’, ‘important’, ‘don’t know/depends’, ‘unimportant’, or ‘should not be included’).

After each survey round, Excel was used to statistically analyse the level of consensus across the three expert panels for each statement. As there is no single definition of consensus across Delphi studies [23], the consensus criteria was based on previous Delphi studies to develop and re-develop mental health first aid guidelines [24–27]. We used the following criteria:

1. Endorsed: The statement was endorsed for inclusion in the guidelines if it received an ‘essential’ or ‘important’ rating from 80 to 100% of panellists from each panel.
2. Re-rate: The statement required re-rating if it received an ‘essential’ or ‘important’ rating from 70 to 79% of panellists from each panel, or an ‘essential’ or ‘important’ rating from 70 to 79% of one or more panels and above 80% from the remaining panels.
3. Rejected: The statement was rejected if it was rated as ‘essential’ or ‘important’ by less than 70% of at least one panel, or if a re-rated new statement (in Round 3) did not receive an ‘essential’ or ‘important’ rating from 80% or more of panellists from each panel.

Following Rounds 1 and 2, panellists were provided with an individualised report on statements that were endorsed, statements that needed to be re-rated in the next survey round, and statements that were rejected. A comparison between each panellist’s individual rating and that of the group was presented in the report for each statement that required re-rating.

As well as re-rate statements, the Round 2 survey included new statements that were developed by the working group from feedback collected from the open-response boxes in the Round 1 survey. The Round 3 survey consisted of the new statements from Round 2 that were neither endorsed nor rejected. Items that did not reach endorsement in Round 3 were rejected from inclusion in the guidelines.

One researcher (JW) analysed areas of disagreement between the three panels. This was done by compiling a list of the statements that were not endorsed due to at least one panel rejecting the statement and one panel endorsing the statement with at least a ± 10% margin between their endorsement ratings. This method has been previously used to discern disagreement between panels in past mental health first aid guideline redevelopment studies that used the Delphi expert consensus method [25, 27]. A comparison between the endorsed and rejected statements from the original Delphi studies and the current Delphi study statements was also undertaken by JW to identify the similarities and differences between the original and redeveloped guidelines.

### Guideline development

The draft of the guidelines was developed by compiling and integrating the statements endorsed by panellists across the three survey rounds into thematic sections, with any repetition removed. The structure and wording of the draft guidelines was revised and finalised by the working group, then disseminated to panellists for any final feedback related to readability and structure. Panellists could not request new content or change existing content at this point.

### Ethics, consent and permissions

This study received ethics approval from the University of Melbourne Human Research Ethics Committee. Informed consent was provided by participants by clicking ‘yes’ to a question in the Round 1 survey.

## Results

### Participants

A total of 119 participants completed the Round 1 survey, with 103 completing all three surveys (see Table 1 for participant characteristics). The retention rates of panellists in each survey round are presented in Table 2. Of the 103 panellists (46 people with lived experience panellists, 33 support people panellists, 24 professional panellists) who completed all three survey rounds, 80 identified as female, 17 identified as male, and 6 identified with another term or did not wish to disclose their gender identity. Panellists were typically aged between 40 and 49 years (range = 18–79) and lived in Australia, Canada, Finland, the Netherlands, New Zealand, and the United Kingdom. The people with lived experience panel included advocates, members of consumer organisations, and lived experience workforce professionals such as consultants, trainers and support workers. The support people panel primarily consisted of advocates with memberships to carer support groups and organisations. The professional panel included senior alcohol and other drug clinicians, researchers, program managers, nurses, social workers, educators, counsellors and support workers, of which 18 panellists also identified as having lived experience (*n* = 2), support person experience (*n* = 15), or both (*n* = 1). 10 panellists also reported having experience as a Mental Health First Aid Instructor.

**Table 1:** Participant characteristics (*n* = 103)

	Age range (years)	Mode age (years)	Gender			Country		
			Female	Male	Other	Australia and New Zealand	Canada	UnitedKingdom and Europe
People with lived experience panel (*n* = 46)	18–79	40–49	30	11	5	28	13	5
Support people panel (*n* = 33)	18–79	60–69	24	2	0	22	9	2
Professional panel (*n* = 24)	30–79	40–49	19	4	1	17	13	4

**Table 2:** Participation of Delphi panelists per panel that completed each round survey

	Round 1	Round 2	Round 3	Retention rate (over 3 rounds)
People with lived experience panel	56	50	46	82%
Support people panel	35	34	33	94%
Professional panel	28	24	24	85%
Total	119	108	103	87%

### Item rating and consensus

**Fig. 2:**
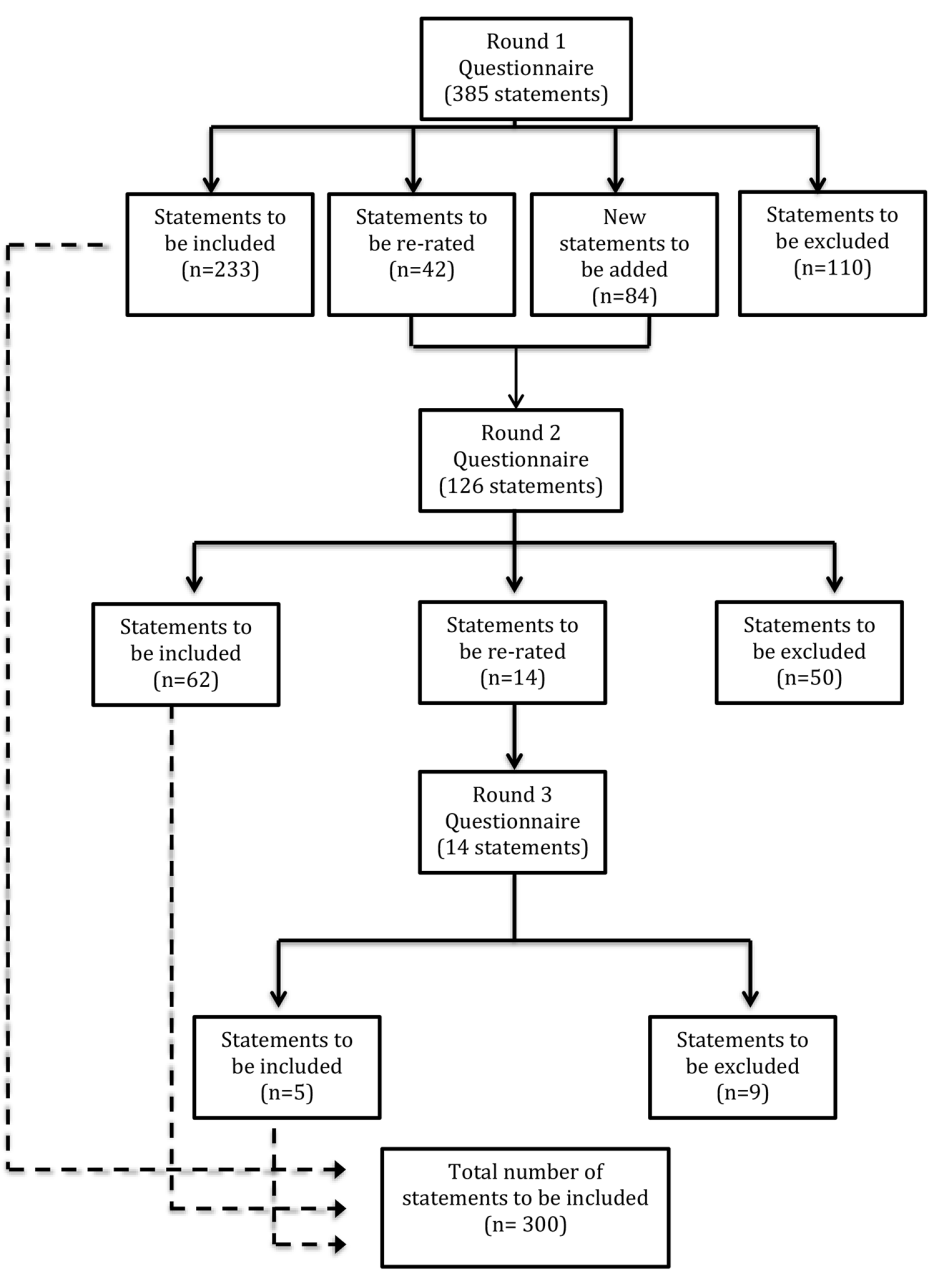
Statements endorsed, re-rated and rejected per survey round

A total of 469 statements were rated across the three survey rounds. 300 items met the 80%+ consensus endorsement criterion for inclusion in the guidelines and 169 items were rejected. The number of statements endorsed, re-rated, and rejected for each survey round is presented in Fig. 2 and the number of statements endorsed and rejected per section of the Delphi questionnaire are presented in Table 3. Pearson’s r was calculated to determine the correlations of endorsement rates across items between the expert panel’s ratings. For the 385 items rated in Round 1, the item endorsement rates between the people with lived experience, support people and professional panels were strongly correlated. Correlation coefficients between the people with lived experience and support people panels, people with lived experience and professional panels, and support people and professional panels were 0.91, 0.91 and 0.92 respectively.

All endorsed and rejected statements over the three survey rounds and their endorsement ratings can be accessed in Additional file 2. The guideline document that was developed from the items endorsed across the three survey rounds is entitled *SUBSTANCE USE PROBLEMS: MHFA GUIDELINES*. It can be accessed in Additional file 3, and is available on the Mental Health First Aid Australia website (https://mhfa.com.au).

**Table 3:** Sections in the Delphi questionnaire and number of statements endorsed and rejected

Section	Topic	Number of items endorsed	Number of items rejected	TOTAL
1	What should the first aider know about substance use problems?	13	0	13
2	When does the person need help?	19	19	38
3	Approaching the person	65	40	105
4	Providing information	17	16	33
5	Supporting the person	64	33	97
6	Supporting someone with a history of substance use problems	13	3	16
7	Professional help	35	11	46
8	Interventions	3	25	28
9	Crisis situations	68	21	89
10	Adolescents	3	1	4
	TOTAL	300	169	469

### Differences between original guidelines and redeveloped guidelines

A total of 300 statements were endorsed in the current guidelines compared to 184 and 140 statements endorsed in the original 2009 and 2011 problem drinking and drug use guidelines respectively. 117 statements from the original guidelines that were included in the redevelopment were re-endorsed for inclusion in the redeveloped guidelines. 10 statements were not re-endorsed for inclusion, e.g., *The first aider should be able to recognise adverse psychological reactions to substances.*

### Differences between the people with lived experience, support people and professional panels

Though most items were rated similarly by the people with lived experience, support people and professional panels, 41 statements were endorsed by one panel and rejected by another, with a difference of at least 10% between the panels. These are presented in Additional file 2.

People with lived experience, support people and professionals rejected eight, seven and seven items respectively, that were endorsed by the other two panels. Support people and professionals rejected nine items that were endorsed by people with lived experience. People with lived experience and professionals rejected five items that were endorsed by the other panel, as did people with lived experience and support people.

There was a lack of consensus between the panels on seven warning signs indicating that a person needs help for a substance use problem, e.g., *The person frequently uses the substance.*

Although all panels endorsed statements relating to providing information and professional help-seeking in Round 1, only items relating to respecting the person’s interest in receiving information and professional help were endorsed by all three panels. Several items recommending the first aider use more direct approaches, such as offering information or encouraging professional help, were rejected by consumers and support people in Round 1. These were later endorsed in Round 2 after the statements were revised to align with panellist feedback that such approaches should only be used if the person is receptive to support (e.g., open to receiving information or interested in professional help).

There was consensus across the panels on the importance of encouraging supports other than professional help, such as reaching out to family and friends, engaging in community activities, and healthy lifestyle changes. Items related to peer support were typically rejected by professionals and support people, despite high endorsement from people with lived experience, e.g., *The first aider should suggest the person connect with other people’s recovery stories, e.g. in-person support groups, consumer events, or online.*

Professionals rejected items in which the first aider suggests professional help when a person is unwilling to change, or the first aider continues to provide social support when the person does not believe they have a problem, e.g., *If the person does not believe they have a problem, the first aider should continue to provide social support.* People with lived experience and professionals endorsed that first aiders should respect a person’s decision to not want professional help, which was rejected by support people.

## Discussion

This study aimed to redevelop and synthesise the original 2009 alcohol and 2011 drug use problem guidelines into mental health first aid guidelines for assisting someone who may have, or may be developing, substance use problems. The three expert panels achieved a high level of consensus on a range of knowledge items and first aid actions, suggesting that people with lived experience, support people and professionals have a similar understanding of what members of the public should know about supporting a person experiencing substance use problems. In total, 300 statements were endorsed by ≥ 80% of panellists and were included in the guidelines. These guidelines provide instructions to first aiders on recognising whether a person may need help for a substance use problem, preparing their approach (including available resources and self-care), how to approach the person and provide them with support and information, encouraging and supporting professional help, what to do when someone does not want to change or professional help, how to support someone experiencing a relapse, and recognising and responding to substance-affected states including medical emergencies and what to do when a person is being aggressive. The substance use guidelines will be made publicly available on the MHFA Australia website (https://mhfa.com.au) and be used to inform future revisions to the MHFA courses.

### The original guidelines vs. the redeveloped guidelines

There was a substantial increase in the number of endorsed statements compared to the previous guidelines. Although the main themes from the original guidelines were retained, as most of the statements included in the redevelopment were re-endorsed for inclusion, the redeveloped guidelines contain more comprehensive detail than the previous guidelines. This suggests there has not been a significant shift in the expert opinions of people with lived experience, support people and professionals on the core set of skills that first aiders should be trained in. It also supports a trend found in other guidelines redevelopment studies and reiterates the importance of updating guidelines regularly to encompass the increase in knowledge and skills available to first aiders [25–27].

The new statements endorsed in the redeveloped guidelines have led to greater specificity on instructions that were minimally covered in the original guidelines, for example, warning signs to indicate the person needs support, when to disclose, providing information and support for harm reduction, and understanding and supporting the person to overcome potential barriers to professional help-seeking. New skills for first aiders to tailor their approach and support depending on the person’s readiness and experiences were also endorsed. These include instructions for providing help to a person who is interested in support and information; does not believe they have a problem; is responsible for children (e.g., pregnant or an infant); is interested in reducing substance-related harm; has a history of substance use problems and experiencing a relapse; or is an adolescent. Unlike the original guidelines, first aiders are also provided with information on confrontational interventions.

### Key discrepancies across panel ratings

Panellists agreed on the inclusion of less than half of the suggested warning signs developed predominantly from websites. This suggests a lack of consensus between people with lived experience, support people and professionals on the important warning signs that indicate a person needs help for a substance use problem. Another explanation is that some warning signs may be specific to some types of substance use problems (e.g., alcohol use only), and not others, with consensus achieved across panels for only warning signs applicable across types more generally. There was also disagreement between the panels on items related to encouraging professional help-seeking and access to support. Unlike professionals, people with lived experience and support people rejected statements that suggested assisting without knowing the person’s willingness regarding, and interests in, help-seeking, information and support. Subsequently, panels agreed to include most of these items after they were adjusted to reflect the importance of the person’s willingness and interests. Disagreement between panels was not similarly resolved for statements for encouraging help-seeking and other supports when a person does not believe they have a problem or are unwilling or reluctant to seek help. These statements were typically endorsed by support people but rejected by professionals and people with lived experience. This discrepancy potentially highlights the lack of support and pressure placed on support people affected by substance use problems, as well as the impact of ongoing substance use problems on support people [30].

Panels also disagreed on the importance of peer support, with people with lived experience endorsing these statements but professionals and support people rejecting them. Despite evidence suggesting that peer support can aid substance use recovery [31],this discrepancy suggests that peer support lacks credibility among professionals and support people.

### Strengths

A key strength of this study was the successful recruitment and retention (between 15 and 30 panellists) [23] of the optimal number of panellists for each panel across all survey rounds, enabling us to explore the degree of consensus between expert groups. Recruitment challenges in previous studies prevented this [24–27, 29]. The inclusion of support people as a distinct panel ensured they were given the same weight of importance in the redevelopment process as people with lived experience and professionals which is valuable given their unique role in prevention and early intervention for substance use problems [32]. Another major strength of this study was that panellists gave suggestions that built upon statements endorsed for inclusion from the original guidelines and literature searches. In doing so, a greater number of first aid actions were identified for inclusion in the guidelines, producing extensive guidelines with new and more comprehensive helping statements that reflect current literature and thinking around substance use (i.e. not treating alcohol as separate from other substances). Also, other mental health first aid guidelines that reference these guidelines (e.g. the suicidal thoughts and behaviours guidelines) [33] will benefit from more comprehensive and up-to-date guidance on supporting someone experiencing or developing a substance use problem. Additionally, having one guideline that covers all substance use problems should make it quicker and simpler for first aiders to find and read important information that is relevant to their situation.

### Limitations

There were several limitations to this study. Although efforts were taken to sample diversely from a range of high-income countries, most panellists were located in Australia. This may have led to biases in responses as mental health systems, professionals and supports differ between countries. However, in Round 1, panellists did have the opportunity to provide open-ended feedback on statements with specific content on professionals or support types that was not available in their country. Some of these statements were revised and re-rated in the following survey round.

These guidelines may not be generalisable to non-Western low and middle-income countries, nor to cultural minorities within Western high income English-speaking countries. However, it is possible the guidelines may be adapted for particular audiences. Recently the Delphi process has been used to adapt guidelines to incorporate actions of cultural importance (e.g. the alcohol guidelines for Argentina, Brazil and China [34–36]) and for cultural minorities within high-income countries (e.g. the Nepalese community in Australia [37]).

A final limitation is that it is currently unclear whether the revised substance use guidelines can be successfully applied by first aiders. Previous research indicates that mental health first aid guidelines, including the original alcohol and drug use guidelines, are downloaded and used by first aiders to support a person experiencing a mental health problem or crisis [38] and research assessing the extent to which first aiders adhere to the guidelines when providing first aid using a recently validated scale is currently underway [39].

## Conclusion

This study demonstrates it is possible to gain consensus across people with lived experience, support people and professionals to redevelop the original guidelines on problem alcohol and drug use guidelines into a broader, more detailed set of first aid guidelines for substance use problems. Using the Delphi method ensures that these guidelines which are used to inform Mental Health First Aid courses, in Australia and internationally, are up to date and provide community members seeking guidance on how to support someone with a substance use problem with the most recent and appropriate helping actions. It is hoped that the additions from the redevelopment will lead to an increase in first aider helping behaviors that appropriately meet the support needs of the individual.

### Electronic supplementary material

Below is the link to the electronic supplementary material.

**Additional file 1**: Surveys for rounds 1–3

**Additional file 2**: Endorsed and rejected statements

**Additional File 3**: Substance use problems: MHFA guidelines

## Data Availability

The datasets used and/or analysed during the current study are available from the corresponding author on reasonable request.
