# Supplementary material for: Redevelopment of mental health first aid guidelines for substance use problems: a Delphi study
Source: BMC Psychol. 2024 Feb 13;12:70. doi: 10.1186/s40359-024-01561-8 (PMC10865545; doi:10.1186/s40359-024-01561-8)
Supplement: Supplementary file 1 — Additional file 1: Surveys for rounds 1–3 [file 40359_2024_1561_MOESM1_ESM.pdf]

Information about this research

. Updating the Mental Health First Aid Guidelines for substance use problems

. Information about this research

. Purpose of this research

Researchers from Mental Health First Aid Australia and The Centre for Mental Health at the University of Melbourne are collaborating to update the mental health first aid guidelines for substance use problems. Mental Health First Aid Australia is a not-for-profit organisation focused on mental health training and research and The Centre for Mental Health is based at the Melbourne School of Population and Global Health at the University of Melbourne.

The aim of this current research project is to update the mental health first aid guidelines for how a member of the public should give assistance to a person who is experiencing substance use problems.

There are current mental health first aid guidelines for alcohol use problems, drug use problems and problem cannabis use that were developed in 2009 (alcohol use problems, drug use problems, problem cannabis). Given that they are over 10 years old, the present study aims to update these guidelines. The guidelines will be available for download on the Mental Health First Aid Australia website (mhfa.com.au) and will be used to inform the Mental Health First Aid course curriculum.

. How we are doing it

The guidelines will be formed on the basis of expert consensus. We are using the consensus of mental health consumers and affected others with advocacy/peer support experience, and mental health professionals, educators and researchers. These experts will complete online surveys to provide their opinions on a range of strategies for how to help someone with problem substance use. The strategies that receive a high level of endorsement will be included in the guidelines.

The strategies to be rated in the surveys are obtained from websites, books, fact sheets, brochures, scientific journal articles and training course materials.

. **What will you be asked to do?**
You will be asked to complete three online surveys over about 4-6 months and the total estimated time commitment is approximately 2-3 hours.

. **What are the possible benefits?**

The guidelines are made publicly available and may benefit your work.

Participants who complete all 3 surveys will be offered an honorarium of AU$200 or equivalent.

. **What are the possible risks?**
We do not anticipate that you will experience any risk or discomfort by participating in this research. In the unlikely event that you become distressed at some stage during your participation, you can contact the crisis support service in your country:

- Australia: Lifeline on 13 11 14
- Canada: National Suicide prevention Lifeline on 1800 273 TALK (8255)
- Denmark: Suicide hotline 70 201 201
- Finland: SOS Crisis Centre 010 195 202
- France: Suicide Écoute 01 45 39 40 00
- Germany: TelephoneSeelsorge 0800/111 0 111
- The Netherlands: Suicide hotline 113Online
- New Zealand: Lifeline Aotearoa on 0800 543 354
- Republic of Ireland: Samaritans on 116 123
- Sweden: Suicide hotline 020 22 00 60
- Switzerland: PARSPAS 027 321 21 21
- UK: Samaritans on 08457 909090
- USA: National Suicide prevention Lifeline on 1800 273 TALK (8255)

If a mental health helpline for your country is not listed here, please visit https://checkpointorg.com/global/, https://www.iasp.info/resources/Crisis_Centres/Europe/ or https://en.wikipedia.org/wiki/List_of_suicide_crisis_lines.

. **If you change your mind**

Participation in this project is voluntary. If you change your mind about participating, you are free to withdraw from the project at any time until the last survey is closed. You may also withdraw your data if you wish, up until the individual survey is closed. Simply contact the research officer (Judith Wright wright.j@unimelb.edu.au).

. **Your Privacy**

Any data we collect from you will be held under password protection and not divulged to others. We are interested in the consensus views of the panels, rather than the views of individual members, so your individual answers will never be reported. We will only present the results in statistical summary form. We occasionally use participant quotes in published journal articles. When this occurs, we do not publish any identifying information with the quote.

Due to research code requirements, we will be storing the information collected for at least 5 years after the study.

. **Who can I contact if I have concerns about this project?**

This research project has been approved by the Human Research Ethics Committee of The University of Melbourne. If you have any concerns or complaints about the conduct of this research project, which you do not wish to discuss with the research team, you should contact the Manager, Human Research Ethics, Research Ethics and Integrity, University of Melbourne, VIC 3010. Tel: +61 3 8344 2073 or Email: HumanEthics- complaints@unimelb.edu.au. All complaints will be treated confidentially. In any correspondence please provide the name of the research team or the name or ethics ID number of the research project. **The ethics ID number of the research project is 1851765.1.**

. **For more information**

You received a Plain Language Statement when you expressed interest in this project (**also available here**). Please refer to this for more details about this study. You may also contact Judith Wright via email for further information: wright.j@unimelb.edu.au.

Consent to participate

. Consent to participate

.

To participate you must be able to read and write English **AND** be 18 years or over **AND**:

- Have a lived experience of problem alcohol or other drug use and consider yourself to be functioning well enough to participate, **AND** be engaged in activities that give you a broader exposure to people's experiences of problem alcohol or other drug use, e.g. you are a member of a consumer advisory or advocacy group, providing peer support to others, etc.

OR

- Have experience in caring for or providing significant day-to-day support to someone with problem alcohol or other drug use **AND** be engaged in activities that give you a broader exposure to people's experiences of problem alcohol or other drug use, e.g. be a member of a carer support group or carer advocacy organisation, etc.

OR

- Be a mental health professional, educator or researcher with at least 5 years' experience working within alcohol and other drug settings.

Q1. Do you meet these criteria?

- ○ Yes
- ○ No (Please exit the survey now.)

Q2.
1. I consent to participate in this project, the details of which have been explained to me, and I have been provided with a **written plain language statement** to keep.
2. I understand that the purpose of this research is to update the Mental Health First Aid Guidelines for problem alcohol and other drug use.
3. I understand that my participation in this project is for research purposes only.

4. I acknowledge that the possible effects of participating in this research project have been explained to my satisfaction.
5. In this project I will be required to complete three online surveys over about 4-6 months.
6. I understand that my participation is voluntary and that I am free to withdraw from this project anytime without explanation or prejudice and to withdraw any unprocessed data that I have provided.
7. I understand that the data from this research will be stored at the University of Melbourne and will be destroyed after 5 years.
8. I have been informed that the confidentiality of the information I provide will be safeguarded subject to any legal requirements; my data will be password protected and accessible only by the named researchers.
9. I understand that given the small number of participants involved in the study, it may not be possible to guarantee my anonymity.
10. I understand that after I consent to participating, my data will be retained by the researchers.

I understand that by submitting this survey I am giving my consent to participate in this study.

- ○ Yes, I understand.
- ○ I do not consent to participating in this research. (Please exit the survey now.)

Instructions

. Instructions

.

Definitions used in this survey

Mental health first aid is the help provided to a person who is developing a mental health problem, experiencing a worsening of an existing mental health problem or in a mental health crisis. The first aid is given until appropriate professional help is received or the crisis resolves.

The person: someone who is experiencing substance use problems.

The first aider: a concerned family member, friend, work colleague or community member, who provides help to a person experiencing substance use problems.

Substances: psychoactive compounds, including alcohol or other drugs. Substances may be legal or illegal.

Substance use problems/Problem substance use: a pattern of harmful use of substances that has the potential to negatively impact a person's physical and mental health, relationships, employment, finances, and the safety of themselves and others. They may or may not have a substance use disorder.

Substance dependence: refers to heavy, regular substance use that results in the person having difficulties controlling the amount they use and experiencing symptoms (such as anxiety) when they stop using or use less than usual. Substance dependence occurs when someone feels they need to use substances in order to get through their day or week.

Withdrawal: refers to a physical condition that occurs when a person, who has been using substances heavily or for extended periods of time, stops using or uses substantially less than usual. Symptoms of withdrawal include severe anxiety, headaches and tremors or shakes. Withdrawal can be a life-threatening condition.

Overdose: is when too much of a substance/s is taken causing harmful effects on the body. Overdoses may be accidental or intentional and can involve substances that are prescription, over-the-counter, legal, or illegal. A person can overdose on many substances, including alcohol (alcohol poisoning), stimulants, pain medication or a mix of substances. An overdose can lead to serious medical complications, including death.

Emergency services: services that respond to and deal with emergencies when they occur, e.g. emergency medical services (ambulance) or law enforcement (the police).

Professional: a broad range of relevantly trained health professionals. This could include a mental health professional, GP/family doctor, hospital emergency staff, ambulance officer or paramedic.

.

Instructions

Please complete the questionnaire by rating each statement according to how important you believe it is for inclusion in the guidelines for providing mental health first aid to someone who is experiencing substance use problems. Please be aware that the statements

in this survey mainly apply to the mental health first aid given to adults. The final section on 'Adolescents' is the one exception.

Please keep in mind that the guidelines will be used by the general public. The statements need to be rated according to their importance for someone WITHOUT a counselling or clinical background.

This questionnaire should take approximately 60-90 minutes to complete. You can complete the survey in two or more sittings. Your answers are saved when you click the ‘Next’ arrow at the bottom of a page. This marks your page and you can begin again at a later date on the next page. Please be aware that once you have logged on and started responding you must complete the questionnaire on the same computer.

In the next phase of the research you will be asked to complete another two surveys over approximately 6 months. The following two surveys will be considerably shorter and take less time to complete.

.

Overview of the questionnaire
Section 1: What should the first aider know about substance use problems?
Section 2: When does the person need help?
Section 3: Approaching the person
Section 4: Providing information
Section 5: Supporting the person
Section 6: Supporting someone with a history of substance use problems
Section 7: Professional help
Section 8: Interventions
Section 9: Crisis situations
Section 10: Adolescents

Information about you

Q16. Information about you

Q17. What is your name? (This allows us to determine who has completed the Round 1 survey and is therefore eligible to participate in Round 2. Your name will be deleted from your data when the project is complete).

Q18. How old are you?

- 18-29
- 30-39
- 40-49
- 50-59
- 60-69
- 70-79
- 80 +

Q19. What is your gender?

- Female
- Male
- I identify with another term
- Prefer not to disclose

Q20. Please indicate your primary source of expertise, i.e. lived experience, support person or professional.

- Person with lived experience
- Support person
- Professional

Q21. In addition to your primary source of expertise, do you also have experience with substance use problems as a:

- Person with lived experience

- ☐ Support person
- ☐ Professional
- ☐ No other experience

Q22. Please state the name of the organisation/s you work or volunteer for that make you eligible to participate in this study.

Q23. What is your role within the above organisation/s?

Q24. What country do you live in?

Q25. Are you a Mental Health First Aid Instructor, i.e. do you deliver the Mental Health First Aid course?

- ○ Yes
- ○ No

What should the first aider know about substance use problems?

Q26. **What should the first aider know about substance use problems?**

This section asks you what information the first aider should know in order to assist the person experiencing substance use problems.

Please rate how important (from 'essential' to 'should not be included') you think it is that each statement be included in the guidelines.

Please also keep the definitions in mind when rating the statements.

Q27. **What are substance use problems?**

Q28. The first aider should not assume that any substance use means the person has a substance use problem, e.g. being dependent.

- Essential
- Important
- Don't know/Depends
- Unimportant
- Should not be included

Q29. The first aider should remember that substance dependence is a mental health problem and not a wilful choice.

- Essential
- Important
- Don't know/Depends
- Unimportant
- Should not be included

Q30. The first aider should be aware that substance use problems are not just a matter of how much of a substance the person is using but how their use affects their life and the lives of those around them.

- Essential
- Important
- Don't know/Depends
- Unimportant
- Should not be included

Q31. **Reasons for use**

Q32. The first aider should have some general knowledge of the range of reasons why people develop substance use problems, e.g. it helps them to cope or stopping leads to unpleasant effects.

- Essential
- Important
- Don't know/Depends
- Unimportant
- Should not be included

Q33. The first aider should be aware that substances are often used to cope with underlying emotional distress or mental illness. This is often called ‘self-medication’.

- Essential
- Important
- Don't know/Depends
- Unimportant
- Should not be included

Q34. **Co-occurring mental health problems**

Q35. The first aider should be aware that mental health problems can be caused or made worse by substance use.

- Essential
- Important
- Don't know/Depends
- Unimportant
- Should not be included

Q36. The first aider should be aware that if the person has underlying emotional distress or mental health problems these may need to be addressed in order for a person to reduce or stop their substance use.

- Essential
- Important
- Don't know/Depends
- Unimportant
- Should not be included

Q37. **Consequences**

Q38. The first aider should be aware of the short- and long- term consequences of substance use problems, including physical, mental or social problems.

- Essential
- Important
- Don't know/Depends
- Unimportant
- Should not be included

Q39. The first aider should be aware of how the substance/s the person is taking may be affecting their behaviour.

- Essential
- Important
- Don't know/Depends
- Unimportant
- Should not be included

Q40. Do you have any comments on the above statements? Is there anything you would like to add? Please write your suggestions in the box provided.

When does the person need help?

Q41. **When does the person need help?**

This section asks you what the first aider should know in order to recognise when the person needs help.

Please rate how important (from 'essential' to 'should not be included') you think it is that each statement be included in the guidelines.

Please also keep the definitions in mind when rating the statements.

Q42. **General recognition**

Q43. The first aider should be able to recognise the signs of substance use problems.

- Essential
- Important
- Don't know/Depends
- Unimportant
- Should not be included

Q44. The first aider should respect the person's privacy, e.g. should not search through their things to look for signs of substance use.

- Essential
- Important
- Don't know/Depends
- Unimportant
- Should not be included

Q45. **Prioritising other's safety**

Q46. If there are any children being affected by the person's substance use, the first aider should make the children’s safety and well-being a priority.

- Essential
- Important
- Don't know/Depends
- Unimportant
- Should not be included

Q47. If the first aider becomes aware that the person’s substance use is placing the safety of others (e.g. partner or family members) at risk, the first aider's priority should be to keep these people safe.

- Essential
- Important
- Don't know/Depends
- Unimportant
- Should not be included

Q48. **Warning signs**

Q49. The first aider should know the following warning signs for recognising substance use problems:

	Essential	Important	Don't know/Depends	Unimportant	Should not be included
*The person is secretive about their substance use.	○	○	○	○	○
*The person plays down how much they use substances.	○	○	○	○	○
*The person is in debt because of the amount	○	○	○	○	○

	Essential	Important	Don't know/Depends	Unimportant	Should not be included
of money they spend on substance use.					
*The person needs to use substances to help deal with certain situations.	○	○	○	○	○
*The person behaves irrationally.	○	○	○	○	○
*The person evades questions about their substance use or looks uncomfortable when responding.	○	○	○	○	○
*The person is unwilling to consider that their substance use is a problem.	○	○	○	○	○
*The person reacts angrily when it is suggested that they have a substance use problem.	○	○	○	○	○
*The person acknowledges they think a lot about substance use and when they'll next get a chance to use.	○	○	○	○	○
*The person appears to prioritise acquiring and using substances over other parts of life, e.g. work and social life.	○	○	○	○	○
*The person has a lack of interest in their personal appearance.	○	○	○	○	○
*The person has a lack of interest in social activities.	○	○	○	○	○
*The person has a lack of interest in previously enjoyed hobbies.	○	○	○	○	○
*The person has become isolated from	○	○	○	○	○

	Essential	Important	Don't know/Depends	Unimportant	Should not be included
both family and friends.					
*The person is neglecting their responsibilities.	○	○	○	○	○
*The person is engaging in criminal conduct, fighting or driving under the influence of the substance.	○	○	○	○	○
*The person frequently uses the substance.	○	○	○	○	○
*The person's lifestyle suddenly changes, e.g. staying out late at night.	○	○	○	○	○
*The person continues their substance use in spite of it causing distress to themselves and the people around them.	○	○	○	○	○
*The person cannot stop or reduce their substance use even if they say they want to.	○	○	○	○	○
*The person spends a lot of time looking for a substance, using it and recovering from using it.	○	○	○	○	○
*The person uses the substance in risky situations, e.g. driving.	○	○	○	○	○
*The person does not recall events.	○	○	○	○	○
*The person uses the substance alone, in the morning or for hours on end.	○	○	○	○	○
*The person uses substances to numb negative feelings or	○	○	○	○	○

	Essential	Important	Don't know/Depends	Unimportant	Should not be included
cope with mental health problems.					

Q50. The first aider should describe the person's substance use behaviour to a professional to see whether they would consider it a problem.

- Essential
- Important
- Don't know/Depends
- Unimportant
- Should not be included

Q51. **Prescription medication**

Q52. The first aider should be aware of the signs the person is misusing a prescription medication.

Signs the person is misusing a prescription medication:

- taking more medication than prescribed or directed on the packet, either in one dose or over time
- taking medication in a different way to what's recommended, such as injecting or snorting
- using medication without a prescription and ongoing medical supervision
- combining it with other drugs, including alcohol
- undertaking activities that medication affects, like driving, working or looking after children
- sharing prescription medication with friends, family or colleagues.

Souce: https://adf.org.au/reducing-risk/pharmaceuticals/

- Essential
- Important
- Don't know/Depends
- Unimportant
- Should not be included

Q53. Do you have any comments on the above statements? Is there anything you would like to add? Please write your suggestions in the box provided.

Approaching the person

Q54. **Approaching the person**

This section asks you how the first aider should approach someone who may be experiencing substance use problems.

Please rate how important (from 'essential' to 'should not be included') you think it is that each statement be included in the guidelines.

Please also keep the definitions in mind when rating the statements.

Q55. **Considerations before making an approach**

Q56. The first aider should be aware that there are a wide range of reasons why people use substances.

- Essential
- Important
- Don't know/Depends
- Unimportant
- Should not be included

Q57. The first aider should be aware that the person may not know why they use substances.

- Essential

- Important
- Don't know/Depends
- Unimportant
- Should not be included

Q58. The first aider should be aware that the environment in which the person uses substances can make it harder or easier for them to change their substance use behaviour.

- Essential
- Important
- Don't know/Depends
- Unimportant
- Should not be included

Q59. The first aider should be aware a person may be reluctant to admit they have a problem for a number of reasons, e.g., due to stigma, the fear of being labelled an 'alcoholic' or an 'addict', effects on relationships.

- Essential
- Important
- Don't know/Depends
- Unimportant
- Should not be included

Q60. **Support for providing mental health first aid**

Q61. The first aider should be aware that they can contact a health professional who specialises in substance use problems to determine how best to approach the person about their concerns.

- Essential
- Important
- Don't know/Depends
- Unimportant

- Should not be included

Q62. The first aider should be aware that they can contact a health professional who specialises in substance use problems for information and support in relation to assisting someone with substance use problems.

- Essential
- Important
- Don't know/Depends
- Unimportant
- Should not be included

Q63. The first aider should be aware that they can consult with others who have dealt with substance use problems about effective ways to help the person.

- Essential
- Important
- Don't know/Depends
- Unimportant
- Should not be included

Q64. The first aider should be aware that they can consult with family and friends to determine how to frame the conversation with the person.

- Essential
- Important
- Don't know/Depends
- Unimportant
- Should not be included

Q65. Before approaching the person, the first aider should find someone who knows the person to help the first aider practice their approach.

- Essential

- Important
- Don't know/Depends
- Unimportant
- Should not be included

Q66. **Specific Planning**

Q67. The first aider should reflect on the person's situation, organise their own thoughts and decide what they want to say to the person.

- Essential
- Important
- Don't know/Depends
- Unimportant
- Should not be included

Q68. The first aider should have some helpful contact numbers with them so the person can call for confidential help or for more information, if they are willing to receive it.

- Essential
- Important
- Don't know/Depends
- Unimportant
- Should not be included

Q69. The first aider should prepare some specific examples of behaviours that they can use to show the person why they are worried.

- Essential
- Important
- Don't know/Depends
- Unimportant
- Should not be included

Q70. Before approaching the person, the first aider should think about their concerns and write them down in a list.

- Essential
- Important
- Don't know/Depends
- Unimportant
- Should not be included

Q71. The first aider should gather information about problem substance use to help them understand and approach the issue.

- Essential
- Important
- Don't know/Depends
- Unimportant
- Should not be included

Q72. If the first aider finds it too difficult to approach the person, they should write a short letter to the person explaining how they feel.

- Essential
- Important
- Don't know/Depends
- Unimportant
- Should not be included

Q73. **Being aware of potential outcomes**

Q74. The first aider should be aware that the person might not believe, or might deny, that they have a substance use problem.

- Essential
- Important
- Don't know/Depends
- Unimportant
- Should not be included

Q75. If the person denies they have a problem, the first aider should be persistent in showing their concern.

- Essential
- Important
- Don't know/Depends
- Unimportant
- Should not be included

Q76. The first aider should be aware that the person may underestimate the amount that they drink or use drugs.

- Essential
- Important
- Don't know/Depends
- Unimportant
- Should not be included

Q77. The first aider should not expect the person to change right away; this conversation may be the first time they have thought of their substance use as a problem.

- Essential
- Important
- Don't know/Depends
- Unimportant
- Should not be included

Q78. The first aider should be aware that the person may find it hard to disclose or discuss their substance use because they are embarrassed or ashamed.

- Essential
- Important
- Don't know/Depends
- Unimportant
- Should not be included

Q79. The first aider should be aware that the person may not recall events that occurred whilst they were intoxicated, i.e. they may have blacked out.

- Essential
- Important
- Don't know/Depends
- Unimportant
- Should not be included

Q80. The first aider should be aware that the person may not want to reduce or stop their substance use.

- Essential
- Important
- Don't know/Depends
- Unimportant
- Should not be included

Q81. The first aider should not expect the person to tell them everything about their substance use.

- Essential
- Important
- Don't know/Depends
- Unimportant

- Should not be included

Q82. The first aider should be aware that that the person may resist the first aider’s help for a number of reasons, e.g. the person might feel that the first aider is trying to take away the person’s “right to drink”.

- Essential
- Important
- Don't know/Depends
- Unimportant
- Should not be included

Q83. The first aider should be aware that the person may give excuses, get angry or try to blame the first aider or other family members for their substance use problem.

- Essential
- Important
- Don't know/Depends
- Unimportant
- Should not be included

Q84. The first aider should be aware that the person may react aggressively when approached about their substance use problem.

- Essential
- Important
- Don't know/Depends
- Unimportant
- Should not be included

Q85. **When to talk**

Q86. The first aider should talk to the person when they are not affected by substances.

- Essential
- Important
- Don't know/Depends
- Unimportant
- Should not be included

Q87. The first aider should approach the person in a setting where there are no substances available or where the person will not be tempted to use or drink.

- Essential
- Important
- Don't know/Depends
- Unimportant
- Should not be included

Q88. The first aider should know how to choose an appropriate time and setting to approach the person.

- Essential
- Important
- Don't know/Depends
- Unimportant
- Should not be included

Q89. The first aider should be aware they may need to have several conversations with the person.

- Essential
- Important
- Don't know/Depends
- Unimportant
- Should not be included

Q90. **Interpersonal skills**

Q91. When talking to the person, the first aider should try to be

	Essential	Important	Don't know/Depends	Unimportant	Should not be included
*calm and reasonable.	○	○	○	○	○
*non-confrontational.	○	○	○	○	○
*assertive but not aggressive.	○	○	○	○	○

Q92. The first aider should know how to engage in non-judgemental communication.

- Essential
- Important
- Don't know/Depends
- Unimportant
- Should not be included

Q93. The first aider should use “I” statements instead of “you” statements, e.g., “I feel worried/angry/frustrated when you…” instead of “You make me feel…”

- Essential
- Important
- Don't know/Depends
- Unimportant
- Should not be included

Q94. The first aider should use concrete examples about how the person's substance use affects the first aider.

- Essential
- Important

- Don't know/Depends
- Unimportant
- Should not be included

Q95. The first aider should use open questions that encourage the person to think about their substance use, e.g. "What do you think about your alcohol use? How do you think you can change it?".

- Essential
- Important
- Don't know/Depends
- Unimportant
- Should not be included

Q96. The first aider should stick to the point (i.e. focus on the person’s substance use) and not get drawn into arguments about other issues.

- Essential
- Important
- Don't know/Depends
- Unimportant
- Should not be included

Q97. The first aider should not criticise the person’s substance use behaviour.

- Essential
- Important
- Don't know/Depends
- Unimportant
- Should not be included

Q98. **What to say and do**

Q99. The first aider should ask the person about their substance use behaviour, e.g. about how much alcohol or drugs the person tends to use.

- Essential
- Important
- Don't know/Depends
- Unimportant
- Should not be included

Q100. The first aider should ask whether the person considers their substance use a problem.

- Essential
- Important
- Don't know/Depends
- Unimportant
- Should not be included

Q101. The first aider should tell the person they will listen without judging them.

- Essential
- Important
- Don't know/Depends
- Unimportant
- Should not be included

Q102. The first aider should try to understand the person’s own perception of their substance use.

- Essential
- Important
- Don't know/Depends
- Unimportant
- Should not be included

Q103. The first aider should try to understand why the person uses substances by asking what they do and don't like about using substances.

- Essential
- Important
- Don't know/Depends
- Unimportant
- Should not be included

Q104. The first aider should ask the person about areas of their life that their substance use may be affecting, e.g. their mood, work performance and relationships.

- Essential
- Important
- Don't know/Depends
- Unimportant
- Should not be included

Q105. The first aider should focus the conversation on the person's behaviour rather than their character, e.g. "Your drinking seems to be getting in the way of your friendships" rather than "You're a useless drunk".

- Essential
- Important
- Don't know/Depends
- Unimportant
- Should not be included

Q106. The first aider should express their concern about how the person is behaving.

- Essential
- Important
- Don't know/Depends

- Unimportant
- Should not be included

Q107. The first aider should explain how they're feeling to the person and how the person's substance use is affecting the first aider.

- Essential
- Important
- Don't know/Depends
- Unimportant
- Should not be included

Q108. The first aider should ask the person if they are aware of the risks associated with substance use problems.

- Essential
- Important
- Don't know/Depends
- Unimportant
- Should not be included

Q109. The first aider should ask the person if they want help to change their substance use behaviour.

- Essential
- Important
- Don't know/Depends
- Unimportant
- Should not be included

Q110. If the person wants help to change their substance use, the first aider should ask what type of help and support they would find most helpful.

- Essential

- Important
- Don't know/Depends
- Unimportant
- Should not be included

Q111. The first aider should offer to help the person and discuss what assistance they are willing to provide.

- Essential
- Important
- Don't know/Depends
- Unimportant
- Should not be included

Q112. The first aider should encourage the person to talk about any problems in their life that may be contributing to their substance use.

- Essential
- Important
- Don't know/Depends
- Unimportant
- Should not be included

Q113. The first aider should reassure the person that they are not alone and that many other people also have problems with substances.

- Essential
- Important
- Don't know/Depends
- Unimportant
- Should not be included

Q114. The first aider should ask the person whether they have made any attempts to change their substance use in the past.

- Essential
- Important
- Don't know/Depends
- Unimportant
- Should not be included

Q115. If the person has previously tried to make a change to their substance use, the first aider should discuss with them what was helpful and what wasn't.

- Essential
- Important
- Don't know/Depends
- Unimportant
- Should not be included

Q116. The first aider should discuss with the person that stopping or reducing substance use is hard, it may be painful, and it takes time.

- Essential
- Important
- Don't know/Depends
- Unimportant
- Should not be included

Q117. The first aider should advise the person that alcohol may interact with other drugs (illicit or prescribed) in an unpredictable way which may lead to a medical emergency.

- Essential
- Important
- Don't know/Depends
- Unimportant

- Should not be included

Q118. The first aider should tell the person that only they can take responsibility for reducing their substance intake.

- Essential
- Important
- Don't know/Depends
- Unimportant
- Should not be included

Q119. The first aider should encourage the person to take responsibility for their actions and deal with consequences in order to achieve long-term change.

- Essential
- Important
- Don't know/Depends
- Unimportant
- Should not be included

Q120. **What to avoid**

Q121. The first aider should be aware that forcing the person to admit they have a substance use problem may cause conflict.

- Essential
- Important
- Don't know/Depends
- Unimportant
- Should not be included

Q122. The first aider should not:

	Essential	Important	Don't know/Depends	Unimportant	Should not be included
*lecture the person.	○	○	○	○	○
*label the person, e.g. by calling them an addict, alcoholic or drunk.	○	○	○	○	○
*press the person to talk if they don't want to talk.	○	○	○	○	○
*try to force the person to admit they have a substance use problem.	○	○	○	○	○
*try to bribe the person to change their substance use.	○	○	○	○	○

Q123. The first aider should avoid:

	Essential	Important	Don't know/Depends	Unimportant	Should not be included
*the use of scare tactics.	○	○	○	○	○
*demonising substances or giving exaggerated messages about substance use.	○	○	○	○	○
*exploring the person's reasons for using substances.	○	○	○	○	○

Q124. The first aider should try to avoid:

	Essential	Important	Don't know/Depends	Unimportant	Should not be included
*making the person feel guilty or ashamed because of their substance use.	○	○	○	○	○
*saying unrealistic things, e.g. "Just don't	○	○	○	○	○

	Essential	Important	Don't know/Depends	Unimportant	Should not be included
drink", "You'll feel better really soon."					
*negating the person's substance use problems , e.g. "Everyone drinks", "You don't look like an addict."	○	○	○	○	○
*glib reassurances, e.g. "Everythings happens for a reason", "Time heals all wounds."	○	○	○	○	○

Q125. If the person does not believe they have a problem, the first aider should:

	Essential	Important	Don't know/Depends	Unimportant	Should not be included
*suggest activities they can do with the person that the person might be responsive to, e.g. reviewing brochures or videos together, both attending a meeting with a professional or a self-help group.	○	○	○	○	○
*let the person know that they are available to talk in the future.	○	○	○	○	○

Q126. **Children**

Q127. If the person is pregnant or breastfeeding, the first aider should:

	Essential	Important	Don't know/Depends	Unimportant	Should not be included
*discuss with them that using substances during pregnancy is unsafe for the baby.	○	○	○	○	○

	Essential	Important	Don't know/Depends	Unimportant	Should not be included
*encourage them to seek appropriate professional help as soon as possible.	○	○	○	○	○
*strongly encourage them to stop using substances while pregnant or breastfeeding.	○	○	○	○	○

Q128. The first aider should encourage the person not to use substances around children.

- Essential
- Important
- Don't know/Depends
- Unimportant
- Should not be included

Q129. If the person is pregnant and has been using substances regularly, the first aider should discuss with the person that suddenly stopping substance use without medical supervision can be dangerous for the unborn baby.

- Essential
- Important
- Don't know/Depends
- Unimportant
- Should not be included

Q130. If the person is pregnant, and wants to stop or reduce their substance use, the first aider should tell them that medical help is essential to do this safely.

- Essential
- Important
- Don't know/Depends

- Unimportant
- Should not be included

Q131. **Encouraging change**

Q132. The first aider should encourage the person to cut down on their substance use.

- Essential
- Important
- Don't know/Depends
- Unimportant
- Should not be included

Q133. The first aider should provide the person with some information about how to cut down on their substance use.

- Essential
- Important
- Don't know/Depends
- Unimportant
- Should not be included

Q134. The first aider should discuss ways the person could use substances without it impacting negatively on others.

- Essential
- Important
- Don't know/Depends
- Unimportant
- Should not be included

Q135. The first aider should discuss ways the person could use substances without it impacting negatively on others.

- Essential
- Important
- Don't know/Depends
- Unimportant
- Should not be included

Q136. **Finding someone who can assist the person**

Q137. If there is a reason that the first aider cannot assist the person effectively, they should help the person find someone else who can.

- Essential
- Important
- Don't know/Depends
- Unimportant
- Should not be included

Q138. Do you have any comments on the above statements? Is there anything you would like to add? Please write your suggestions in the box provided.

Providing information

Q139. **Providing information**

This section asks you what information the first aider should provide to the person.

Please rate how important (from 'essential' to 'should not be included') you think it is that each statement be included in the guidelines.

Please also keep the **definitions** in mind when rating the statements.

Q140. **What information should the first aider provide?**

Q141. The first aider should provide some basic facts about substance use problems, e.g. how common they are, the associated risks, available treatments.

- Essential
- Important
- Don't know/Depends
- Unimportant
- Should not be included

Q142. The first aider should offer the person some information about substance use problems.

- Essential
- Important
- Don't know/Depends
- Unimportant
- Should not be included

Q143. The first aider should be aware that the person may already know a lot about substance use problems.

- Essential
- Important
- Don't know/Depends
- Unimportant
- Should not be included

Q144. The first aider should help the person get some information on how to change their problem substance use.

- Essential
- Important
- Don't know/Depends
- Unimportant
- Should not be included

Q145. The first aider should discuss with the person some risks associated with problem substance use.

- Essential
- Important
- Don't know/Depends
- Unimportant
- Should not be included

Q146. The first aider should offer to help the person get information on:

	Essential	Important	Don't know/Depends	Unimportant	Should not be included
*online screening questionnaires.	O	O	O	O	O
*a range of local treatment options and allow the person to decide which would be most appropriate or useful for them.	O	O	O	O	O
*information about self-help strategies, e.g. reading books about changing substance use, or attending a support group.	O	O	O	O	O

Q147. The first aider should encourage the person to use a self-help method or join a support group program, e.g. Alcoholics Anonymous (AA).

- Essential

- Important
- Don't know/Depends
- Unimportant
- Should not be included

Q148. The first aider should have some knowledge of local, state and national laws around substance use and possession.

- Essential
- Important
- Don't know/Depends
- Unimportant
- Should not be included

Q149. The first aider should have some knowledge about cultural norms around substance use for the person they are assisting.

- Essential
- Important
- Don't know/Depends
- Unimportant
- Should not be included

Q150. **Harm minimisation**

Q151. The first aider should be aware that there are some ways of using substances that are less harmful than other ways.

- Essential
- Important
- Don't know/Depends
- Unimportant
- Should not be included

Q152. The first aider should tell the person that cutting back on their use of substances may be helpful.

- Essential
- Important
- Don't know/Depends
- Unimportant
- Should not be included

Q153. The first aider should tell the person that some people are able to cut back and use small amount of substance in the future without any problems.

- Essential
- Important
- Don't know/Depends
- Unimportant
- Should not be included

Q154. The first aider should encourage the person to find some information on how to reduce the harms associated with their problem substance use.

- Essential
- Important
- Don't know/Depends
- Unimportant
- Should not be included

Q155. The first aider should provide the person with information about harm reduction strategies.

- Essential
- Important
- Don't know/Depends

- Unimportant
- Should not be included

Q156. If the person's substance use problems include alcohol:

	Essential	Important	Don't know/Depends	Unimportant	Should not be included
*the first aider should be familiar with any national drinking guidelines on reducing health risks.	O	O	O	O	O
*the first aider should ask the person if they would like some tips on reducing their health risks when drinking. *^^^^	O	O	O	O	O

Q157.

***^^^^ PRACTICAL TIPS FOR REDUCING HEALTH RISKS WHEN DRINKING**

• Know how much alcohol is in a standard drink

• Know the number of standard drinks in each beverage (the number of standard drinks is often listed on the beverage's packaging)

• Keep count of the number of standard drinks consumed

• Do not let people top up your glass before it is finished, so as not to lose track of how much alcohol has been consumed

• Eat while drinking

• Drink plenty of water when drinking alcohol to prevent dehydration

• Drink beverages with lower alcohol content (e.g. low-alcohol beer instead of fullstrength beer)

• Switch to non-alcoholic drinks when starting to feel the effects of alcohol

• Avoid keeping up with friends drink for drink

• Avoid drinking competitions and drinking games

• Drink slowly, for example, by taking sips instead of gulps and putting the drink down between sips

• Only have one drink at a time

• Spend time on activities that don't involve drinking

• Drink alcohol as part of another activity instead of making it the main activity

• Identify situations where drinking is likely and avoid them if possible

Q158. If the person is injecting drugs, the first aider should:

	Essential	Important	Don't know/Depends	Unimportant	Should not be included
*tell the person that it is never ok to share injecting equipment (needles, syringes, tourniquets, filters, spoons, waters) with someone else, not even if they say they are clean, if they are family, or if the person knows them really well.	O	O	O	O	O
*tell the person how they can find out about harm reduction programs, e.g. needle exchange programs, safe injecting rooms.	O	O	O	O	O

Q159. Do you have any comments on the above statements? Is there anything you would like to add? Please write your suggestions in the box provided.

Supporting the person

Q160. **Supporting the person**

This section asks you how the first aider should support the person.

Please rate how important (from 'essential' to 'should not be included') you think it is that each statement be included in the guidelines.

Please also keep the **definitions** in mind when rating the statements.

Q161. **How to support the person if they are unwilling to change**

Q162. If the person is unwilling to change their substance use, the first aider should:

	Essential	Important	Don't know/Depends	Unimportant	Should not be included
*give the person a clear message that changing their substance use is the best option.	○	○	○	○	○
*tell the person they are concerned about their continuing substance use.	○	○	○	○	○
*tell the person that substance use is never completely safe.	○	○	○	○	○
*be aware that if they continue to be supportive they may be able to help the person change their problem substance use in the future.	○	○	○	○	○
*explain the consequences of continuing with their current substance use behaviours.	○	○	○	○	○
*be patient while waiting for the person to accept they have a problem.	○	○	○	○	○
*assure the person that the first aider will be there if the person wants help or to talk again in the future.	○	○	○	○	○
*tell the person what behaviour they are willing to accept from the person, e.g. the	○	○	○	○	○

	Essential	Important	Don't know/Depends	Unimportant	Should not be included
first aider won't accept the person coming to the first aider's home for a social visit when they are intoxicated.					

Q163. The first aider should discuss with the person that using substances to escape problems just makes the problems worse.

- Essential
- Important
- Don't know/Depends
- Unimportant
- Should not be included

Q164. If the person is unwilling to change their substance use, the first aider should not:

	Essential	Important	Don't know/Depends	Unimportant	Should not be included
*try to control the person by bribing, nagging, threatening or crying.	○	○	○	○	○
*take on the person's responsibilities except if not doing so would cause harm, e.g. to their own or other's lives.	○	○	○	○	○
*deny the person basic needs.	○	○	○	○	○
*cover up or make excuses for the person.	○	○	○	○	○
*use substances with the person.	○	○	○	○	○
*feel guilty or responsible for the person's decision to	○	○	○	○	○

	Essential	Important	Don't know/Depends	Unimportant	Should not be included
keeping using substances.					
*provide the person with money to buy substances.	○	○	○	○	○
*get involved with helping the person obtain substances, e.g. driving the person to meet a dealer.	○	○	○	○	○
*use negative approaches such as lecturing the person or making them feel guilty as these are unlikely to promote change.	○	○	○	○	○

Q165. Do you have any comments on the above statements? Is there anything you would like to add? Please write your suggestions in the box provided.

Supporting the person (continued)

Q166. **Supporting the person (continued)**

This section asks you how the first aider should support the person.

Please rate how important (from ‘essential’ to ‘should not be included’) you think it is that each statement be included in the guidelines.

Please also keep the **definitions** in mind when rating the statements.

Q167. **How to support the person if they are willing to change**

Q168. **Understanding change**

Q169. The first aider should be aware that changing problem substance use is a process that takes time.

- Essential
- Important
- Don't know/Depends
- Unimportant
- Should not be included

Q170. The first aider should know that trying to cut back on substance use is hard, and the person may experience emotional and physical stress.

- Essential
- Important
- Don't know/Depends
- Unimportant
- Should not be included

Q171. The first aider should be aware that it is possible for the person to change their substance use.

- Essential
- Important
- Don't know/Depends
- Unimportant
- Should not be included

Q172. The first aider should be aware that substances can interfere with the person's normal thought processes making it difficult for the person to understand that change is necessary.

- Essential
- Important

- Don't know/Depends
- Unimportant
- Should not be included

Q173. The first aider should know that even doing small things to help can make a difference to the person's substance use problems.

- Essential
- Important
- Don't know/Depends
- Unimportant
- Should not be included

Q174. The first aider should be aware that the person is the only one who can make the decision to change their substance use behaviour.

- Essential
- Important
- Don't know/Depends
- Unimportant
- Should not be included

Q175. The first aider should be aware that a person's willpower is not always enough to help them overcome substance use problems.

- Essential
- Important
- Don't know/Depends
- Unimportant
- Should not be included

Q176. The first aider should be aware that detoxification is only part of recovery and lifestyle changes are required to change substance use behaviours.

- Essential
- Important
- Don't know/Depends
- Unimportant
- Should not be included

Q177. The first aider should be aware that it is not easy to change substance use habits.

- Essential
- Important
- Don't know/Depends
- Unimportant
- Should not be included

Q178. The first aider should be aware that changing habits takes time and repeated efforts.

- Essential
- Important
- Don't know/Depends
- Unimportant
- Should not be included

Q179. The first aider should be aware that giving advice alone may not help the person change their substance use behaviour.

- Essential
- Important
- Don't know/Depends
- Unimportant
- Should not be included

Q180. The first aider should be aware that they cannot force the person to accept help if

they are not ready to do so.

- Essential
- Important
- Don't know/Depends
- Unimportant
- Should not be included

Q181. The first aider should be aware that if they maintain a good relationship with the person they may be able to have a beneficial effect on the person’s substance use problems.

- Essential
- Important
- Don't know/Depends
- Unimportant
- Should not be included

Q182. **Supporting change**

Q183. If the person wants to stop or reduce their substance use, the first aider should:

	Essential	Important	Don't know/Depends	Unimportant	Should not be included
*inform the person of the range of supports that are available.	○	○	○	○	○
*allow the person to decide which supports are most appropriate or useful for them.	○	○	○	○	○
*encourage the person to talk to someone they trust, e.g. a friend, family member or community support worker.	○	○	○	○	○
*encourage the person to seek professional advice before they	○	○	○	○	○

	Essential	Important	Don't know/Depends	Unimportant	Should not be included
change their substance use behaviour.					

Q184. The first aider should be aware that if the person has been drinking or using heavily for a long time, stopping suddenly can be very dangerous to their health.

- Essential
- Important
- Don't know/Depends
- Unimportant
- Should not be included

Q185. The first aider should encourage the person to seek medical help if the person has been drinking or using heavily for long periods and decides to stop suddenly.

- Essential
- Important
- Don't know/Depends
- Unimportant
- Should not be included

Q186. The first aider should be positive and encouraging of any efforts the person makes to change their problem substance use.

- Essential
- Important
- Don't know/Depends
- Unimportant
- Should not be included

Q187. The first aider should encourage the person to keep trying if they have a setback.

- Essential
- Important
- Don't know/Depends
- Unimportant
- Should not be included

Q188. The first aider should help the person stay focused on positive goals that do not involve substances.

- Essential
- Important
- Don't know/Depends
- Unimportant
- Should not be included

Q189. The first aider should support and acknowledge the positive things the person does and achieves.

- Essential
- Important
- Don't know/Depends
- Unimportant
- Should not be included

Q190. The first aider should support and encourage any small changes or improvements in the person's substance use behaviour.

- Essential
- Important
- Don't know/Depends
- Unimportant
- Should not be included

Q191. The first aider should encourage the person to reach out to friends and family who support their efforts to change their substance use.

- Essential
- Important
- Don't know/Depends
- Unimportant
- Should not be included

Q192. The first aider should warn the person that not all family and friends will be supportive of their efforts to change their substance use.

- Essential
- Important
- Don't know/Depends
- Unimportant
- Should not be included

Q193. The first aider should encourage the person to find ways of coping when they feel the urge to use substances, e.g. talking to someone they trust, participating in family or community activities.

- Essential
- Important
- Don't know/Depends
- Unimportant
- Should not be included

Q194. The first aider should encourage the person to talk to a friend or someone they trust about their problems, not just their substance use.

- Essential
- Important
- Don't know/Depends

- Unimportant
- Should not be included

Q195. The first aider should encourage the person to speak to people who have stopped using substances.

- Essential
- Important
- Don't know/Depends
- Unimportant
- Should not be included

Q196. The first aider should encourage the person to spend time with family or friends that don't use substances.

- Essential
- Important
- Don't know/Depends
- Unimportant
- Should not be included

Q197. Do you have any comments on the above statements? Is there anything you would like to add? Please write your suggestions in the box provided.

Supporting the person (continued)

Q198. **Supporting the person (continued)**

This section asks you how the first aider should support the person.

Please rate how important (from 'essential' to 'should not be included') you think it is that each statement be included in the guidelines.

Please also keep the **definitions** in mind when rating the statements.

Q199. **Providing other supports**

Q200. **Encouraging other supports**

Q201. The first aider should offer to help the person find support from others who have had personal experience of substance use problems and successful recovery.

- Essential
- Important
- Don't know/Depends
- Unimportant
- Should not be included

Q202. The first aider should know that trying to cut back on substance use is hard, and the person may experience emotional and physical stress.

- Essential
- Important
- Don't know/Depends
- Unimportant
- Should not be included

Q203. The first aider should encourage the person not to push away the people who worry or complain about their substance use, as these people are a potential source of support.

- Essential
- Important
- Don't know/Depends
- Unimportant

- Should not be included

Q204. The first aider should support the positive choices that the person makes, e.g. playing sport or spending time with people that don't use substances.

- Essential
- Important
- Don't know/Depends
- Unimportant
- Should not be included

Q205. The first aider should encourage the person to take up a hobby or new interest to take their mind off substance use.

- Essential
- Important
- Don't know/Depends
- Unimportant
- Should not be included

Q206. **Managing social pressure**

Q207. The first aider should have an understanding of the different substances used in the person's community.

- Essential
- Important
- Don't know/Depends
- Unimportant
- Should not be included

Q208. The first aider should encourage the person to be strong and say no when they don't

want to take substances.

- Essential
- Important
- Don't know/Depends
- Unimportant
- Should not be included

Q209. The first aider should work with the person to think of ways to say no when offered substances, e.g. “I don’t feel like it”, "I don't feel well", "I am taking medication” or volunteer to be the designated driver.

- Essential
- Important
- Don't know/Depends
- Unimportant
- Should not be included

Q210. The first aider should reassure the person that saying no to substances will get easier the more they do it.

- Essential
- Important
- Don't know/Depends
- Unimportant
- Should not be included

Q211. The first aider should reassure the person that they do not need to explain to others why they are not using substances.

- Essential
- Important
- Don't know/Depends
- Unimportant

- Should not be included

Q212. The first aider should suggest the person try to stay away from people who pressure them to use substances.

- Essential
- Important
- Don't know/Depends
- Unimportant
- Should not be included

Q213. The first aider should suggest that, when under pressure to drink, the person can always pour themselves a soft drink and let others assume it is a mixed drink.

- Essential
- Important
- Don't know/Depends
- Unimportant
- Should not be included

Q214. The first aider should tell the person that the people who care about them will accept their decision not to use.

- Essential
- Important
- Don't know/Depends
- Unimportant
- Should not be included

Q215. **Providing support with boundaries**

Q216. The first aider should find ways to support the person without supporting their

substance use, e.g. buy the person food rather than giving them money.

- Essential
- Important
- Don't know/Depends
- Unimportant
- Should not be included

Q217. The first aider should communicate a sense of hope and belief that the person can change their problem substance use.

- Essential
- Important
- Don't know/Depends
- Unimportant
- Should not be included

Q218. The first aider should not make excuses or cover up the behaviour of the person to protect them from the consequences of their substance use.

- Essential
- Important
- Don't know/Depends
- Unimportant
- Should not be included

Q219. The first aider should establish boundaries of acceptable and unacceptable behaviour with the person.

- Essential
- Important
- Don't know/Depends
- Unimportant
- Should not be included

Q220. The first aider should discuss with the person the level of support they are willing to provide.

- Essential
- Important
- Don't know/Depends
- Unimportant
- Should not be included

Q221. If the person refuses the first aider's help, the first aider should make sure the person is aware of the consequences of making that decision, e.g. they cannot meet with the first aider while under the influence of substances.

- Essential
- Important
- Don't know/Depends
- Unimportant
- Should not be included

Q222. **Healthy lifestyle**

Q223. The first aider should encourage the person to eat healthy so their body can be strong while they are trying to change their problem substance use.

- Essential
- Important
- Don't know/Depends
- Unimportant
- Should not be included

Q224. The first aider should encourage the person to try and get a good amount of sleep so their body can work to repair itself while they are trying to change their problem substance

use.

- Essential
- Important
- Don't know/Depends
- Unimportant
- Should not be included

Q225. The first aider should encourage the person to find healthy ways to feel good instead of using substances.

- Essential
- Important
- Don't know/Depends
- Unimportant
- Should not be included

Q226. The first aider should encourage the person to do more of what keeps them feeling well, e.g. connecting with family or friends, enjoying favourite activities.

- Essential
- Important
- Don't know/Depends
- Unimportant
- Should not be included

Q227. **Disclosure**

Q228. The first aider should not disclose the person's problem substance use to a **friend or family member** unless:

	Essential	Important	Don't know/Depends	Unimportant	Should not be included
*they have the person's consent.	○	○	○	○	○

	Essential	Important	Don't know/Depends	Unimportant	Should not be included
*the person is a child or young person.	○	○	○	○	○
*the person is at risk of harm to themselves.	○	○	○	○	○
*the person is at risk of harming others.	○	○	○	○	○
*there is a child or young person being placed at risk because of the person’s problem substance use.	○	○	○	○	○

Q229. The first aider should not disclose the person’s problem substance use to a **professional** unless:

	Essential	Important	Don't know/Depends	Unimportant	Should not be included
*they have the person’s consent.	○	○	○	○	○
*the person is a child or young person.	○	○	○	○	○
*the person is at risk of harm to themselves.	○	○	○	○	○
*the person is at risk of harming others.	○	○	○	○	○
*there is a child or young person being placed at risk because of the person’s problem substance use.	○	○	○	○	○

Q230. **Self-care for the first aider**

Q231. If the first aider is feeling upset, overwhelmed, stressed or unwell after helping the person, they should use self-care strategies.

- Essential
- Important
- Don't know/Depends
- Unimportant
- Should not be included

Q232. The first aider should be aware that their own self-care is as important as the care offered to the person.

- Essential
- Important
- Don't know/Depends
- Unimportant
- Should not be included

Q233. The first aider should access informal and formal help sources themselves, and on behalf of the person, as then they will be more likely to maintain their important support-giving role.

- Essential
- Important
- Don't know/Depends
- Unimportant
- Should not be included

Q234. The first aider should be aware that alcohol and drug helplines are often an available support option ideal for supporters of people with substance use problems who feel stigmatised or prefer to remain anonymous.

- Essential
- Important
- Don't know/Depends
- Unimportant
- Should not be included

Q235. Do you have any comments on the above statements? Is there anything you would like to add? Please write your suggestions in the box provided.

Supporting someone with a history of substance use problems

Q236. **Supporting someone with a history of substance use problems**

This section asks you how the first aider should support someone with a prior history of substance use problems.

Please rate how important (from 'essential' to 'should not be included') you think it is that each statement be included in the guidelines.

Please also keep the **definitions** in mind when rating the statements.

Q237. The first aider should be aware that the person may relapse once or several times before changing their substance use patterns.

- Essential
- Important
- Don't know/Depends
- Unimportant
- Should not be included

Q238. The first aider should know the stages of change and the process it takes for somebody to change their behaviour.

- Essential
- Important
- Don't know/Depends

- Unimportant
- Should not be included

Q239. The first aider should be aware of the different types of help needed at each stage of change.

- Essential
- Important
- Don't know/Depends
- Unimportant
- Should not be included

Q240. The first aider should know how to help a person who has relapsed.

- Essential
- Important
- Don't know/Depends
- Unimportant
- Should not be included

Q241. The first aider should develop a clear, written plan for what to do in the case of an emergency related to the person's substance use, and, if required, involve a mental health professional in developing the plan.

- Essential
- Important
- Don't know/Depends
- Unimportant
- Should not be included

Q242. The first aider should be aware that if the intoxicated person needs to be contained, sobering up shelters and drug and alcohol resource centres are preferable to police lock-

ups, because they can help the person stay safe, learn about their drinking and its risks, and get some professional help.

- Essential
- Important
- Don't know/Depends
- Unimportant
- Should not be included

Q243. If the person has previously tried a treatment that didn't work, the first aider should encourage them to try seeking professional help again because new options may be available.

- Essential
- Important
- Don't know/Depends
- Unimportant
- Should not be included

Q244. The first aider should know that it may take some time to find a health professional with whom the person is able to establish a good relationship. If this is the case, the first aider should encourage the person not to give up.

- Essential
- Important
- Don't know/Depends
- Unimportant
- Should not be included

Q245. Do you have any comments on the above statements? Is there anything you would like to add? Please write your suggestions in the box provided.

Professional help

Q246. **Professional help**

This section asks you how the first aider should connect the person with professional help.

Please rate how important (from 'essential' to 'should not be included') you think it is that each statement be included in the guidelines.

Please also keep the ***definitions*** in mind when rating the statements.

Q247. **Approaching the person about professional help**

Q248. The first aider should ask the person if they would like to get professional help.

- Essential
- Important
- Don't know/Depends
- Unimportant
- Should not be included

Q249. The first aider should encourage the person to seek professional help.

- Essential
- Important
- Don't know/Depends
- Unimportant
- Should not be included

Q250. The first aider should discuss with the person the benefits of professional help.

- Essential
- Important
- Don't know/Depends

- Unimportant
- Should not be included

Q251. The first aider should be aware that it is common for people with substance use problems to resist seeking professional help.

- Essential
- Important
- Don't know/Depends
- Unimportant
- Should not be included

Q252. The first aider should be aware that there are effective interventions for substance use problems.

- Essential
- Important
- Don't know/Depends
- Unimportant
- Should not be included

Q253. The first aider should be aware that the sooner professional help commences, the better the person's chances for recovery.

- Essential
- Important
- Don't know/Depends
- Unimportant
- Should not be included

Q254. The first aider should be aware of the range of treatment options and support services available such as education, counselling, therapy, rehabilitation and self-help groups.

- Essential

- Important
- Don't know/Depends
- Unimportant
- Should not be included

Q255. The first aider should be aware of what treatment options are available in the person's local area.

- Essential
- Important
- Don't know/Depends
- Unimportant
- Should not be included

Q256. The first aider should be aware that some treatment programs aim to have a substance-free lifestyle, while other programs aim to reduce the person's substance use.

- Essential
- Important
- Don't know/Depends
- Unimportant
- Should not be included

Q257. If the person is experiencing any symptoms of mental illness, the first aider should encourage the person to seek professional help, whether the symptoms are substance-related or not.

- Essential
- Important
- Don't know/Depends
- Unimportant
- Should not be included

Q258. The first aider should encourage the person to call emergency services if they have an adverse reaction while using substances.

- Essential
- Important
- Don't know/Depends
- Unimportant
- Should not be included

Q259. **Encouraging professional help**

Q260. The first aider should explain to the person that there are several treatment approaches available.

- Essential
- Important
- Don't know/Depends
- Unimportant
- Should not be included

Q261. The first aider should not pressure the person into a single type of treatment.

- Essential
- Important
- Don't know/Depends
- Unimportant
- Should not be included

Q262. The first aider should be prepared for the person to respond negatively when professional help is suggested.

- Essential
- Important
- Don't know/Depends

- Unimportant
- Should not be included

Q263. The first aider should not use a confrontational or coercive approach when trying to get the person to seek professional help.

- Essential
- Important
- Don't know/Depends
- Unimportant
- Should not be included

Q264. The first aider should tell the person that it takes courage to ask for, and to accept, professional help.

- Essential
- Important
- Don't know/Depends
- Unimportant
- Should not be included

Q265. The first aider should give the person information about local options for professional help.

- Essential
- Important
- Don't know/Depends
- Unimportant
- Should not be included

Q266. The first aider should offer to accompany the person to appointments with professionals.

- Essential

- Important
- Don't know/Depends
- Unimportant
- Should not be included

Q267. If the person seeks professional help, the first aider should follow up with the person to see how the appointment went.

- Essential
- Important
- Don't know/Depends
- Unimportant
- Should not be included

Q268. If the person does not want to get help for their substance use problems, the first aider should encourage the person to get a medical check-up.

- Essential
- Important
- Don't know/Depends
- Unimportant
- Should not be included

Q269. If the person is reluctant to seek professional help for their substance use problem, the first aider should encourage them to seek help for any other impacts that substance use is having on their lives, e.g. relationship problems, aggression or financial problems.

- Essential
- Important
- Don't know/Depends
- Unimportant
- Should not be included

Q270. **If the person is unwilling to seek professional help**

Q271. The first aider should be aware that the person cannot be forced to get professional help except under certain circumstances, e.g. if a violent incident results in the police being called or following a medical emergency.

- Essential
- Important
- Don't know/Depends
- Unimportant
- Should not be included

Q557. The first aider should be aware that the person cannot be forced to get professional help except under certain circumstances, e.g. if a violent incident results in the police being called or following a medical emergency.

- Essential
- Important
- Don't know/Depends
- Unimportant
- Should not be included

Q272. If the person is unwilling to seek professional help, the first aider should:

	Essential	Important	Don't know/Depends	Unimportant	Should not be included
*not use negative approaches to get the person to seek professional help such as threats, accusations, nagging or ridiculing.	○	○	○	○	○
*be aware that it is ultimately the person’s decision to get professional help.	○	○	○	○	○

	Essential	Important	Don't know/Depends	Unimportant	Should not be included
*be prepared to talk to the person again in the future.	○	○	○	○	○
*be patient and remain optimistic because opportunities will present themselves to suggest professional help again.	○	○	○	○	○
*continue to suggest professional help to the person if they are putting themselves or others at risk of harm.	○	○	○	○	○

Q273. If the person is unwilling to seek in-person professional help, the first aider should suggest telehealth services as an option.

- ○ Essential
- ○ Important
- ○ Don't know/Depends
- ○ Unimportant
- ○ Should not be included

Q274. The first aider should be aware that the person may only accept professional help when the consequences of their substance use become serious enough.

- ○ Essential
- ○ Important
- ○ Don't know/Depends
- ○ Unimportant
- ○ Should not be included

Q275. If the person does not want professional help, the first aider should respect their decision.

- Essential
- Important
- Don't know/Depends
- Unimportant
- Should not be included

Q276. The first aider should not feel like they have failed if the person does not seek professional help.

- Essential
- Important
- Don't know/Depends
- Unimportant
- Should not be included

Q277. The first aider should give the person a card or phone number of a service they can use when they feel ready.

- Essential
- Important
- Don't know/Depends
- Unimportant
- Should not be included

Q278. The first aider should be compassionate and patient while waiting for the person to accept they need help.

- Essential
- Important
- Don't know/Depends
- Unimportant
- Should not be included

Q279. **Addressing barriers and supporting help-seeking**

Q280. The first aider should explain to the person that you don’t need to be an ‘alcoholic’ or 'addict' to benefit from talking to a professional.

- Essential
- Important
- Don't know/Depends
- Unimportant
- Should not be included

Q281. The first aider should explain that the goal of professional help may be to help the person to find ways to reduce their substance use, rather than to make the person quit altogether.

- Essential
- Important
- Don't know/Depends
- Unimportant
- Should not be included

Q282. The first aider should reassure the person that professional help is confidential.

- Essential
- Important
- Don't know/Depends
- Unimportant
- Should not be included

Q283. The first aider should tell the person that there is no shame in getting help to change their problem substance use.

- Essential
- Important

- Don't know/Depends
- Unimportant
- Should not be included

Q284. Do you have any comments on the above statements? Is there anything you would like to add? Please write your suggestions in the box provided.

Interventions

Q285. **Interventions**

This section asks you what the first aider should know about interventions.

Please rate how important (from 'essential' to 'should not be included') you think it is that each statement be included in the guidelines.

Please also keep the **definitions** in mind when rating the statements.

Intervention definition: *a group of people coming together to confront a person about their substance use.*

Q286. The first aider should not organise an intervention.

- Essential
- Important
- Don't know/Depends
- Unimportant
- Should not be included

Q287. The first aider should consult a health professional before carrying out an

intervention.

- Essential
- Important
- Don't know/Depends
- Unimportant
- Should not be included

Q288. The first aider should be aware that the goal of an intervention is to:

	Essential	Important	Don't know/Depends	Unimportant	Should not be included
*make the person aware of the negative consequences of the substance use problems.	○	○	○	○	○
*encourage the person to get professional help.	○	○	○	○	○
*get the person to agree to enter into a treatment program.	○	○	○	○	○

Q289. The first aider should be aware there is no evidence to suggest that confrontational interventions effectively convince people they have a problem or motivate them to change.

- Essential
- Important
- Don't know/Depends
- Unimportant
- Should not be included

Q290. **Planning an intervention**

Q291. The first aider should carry out an intervention if the person:

	Essential	Important	Don't know/Depends	Unimportant	Should not be included
*is unwilling to discuss their substance use problems.	○	○	○	○	○
*continues to make excuses for their erratic behaviours.	○	○	○	○	○
*refuses to admit they have a problem.	○	○	○	○	○
*is unwilling to seek professional help.	○	○	○	○	○

Q292. If the first aider is carrying out an intervention they should:

	Essential	Important	Don't know/Depends	Unimportant	Should not be included
*plan it well beforehand.	○	○	○	○	○
*choose a time and place when the person is least likely to be affected by substances.	○	○	○	○	○

Q293. The first aider should not let the person know about the intervention beforehand.

- ○ Essential
- ○ Important
- ○ Don't know/Depends
- ○ Unimportant
- ○ Should not be included

Q294. Before running an intervention with the person, the first aider should:

	Essential	Important	Don't know/Depends	Unimportant	Should not be included
*gather information about the extent of the person's problem.	○	○	○	○	○

	Essential	Important	Don't know/Depends	Unimportant	Should not be included
*research the person's type of problem and treatment programs.	○	○	○	○	○
*initiate arrangements to enrol the person in a specific treatment program.	○	○	○	○	○

Q295. If the first aider is carrying out an intervention they should:

	Essential	Important	Don't know/Depends	Unimportant	Should not be included
*present the person with different options for professional treatment.	○	○	○	○	○
*form an intervention team of people who will personally participate in the intervention.	○	○	○	○	○
*make sure that everyone that will be involved in the intervention has the same information about the person's substance use and what will happen at the intervention.	○	○	○	○	○
*hold a rehearsal intervention with those that will be involved and decide details of how the intervention will run.	○	○	○	○	○

Q296. The first aider should include people that are important in the person's life, e.g. friends and family.

○ Essential

- Important
- Don't know/Depends
- Unimportant
- Should not be included

Q297. **During the intervention**

Q298. During the planned intervention, the first aider should:

	Essential	Important	Don't know/Depends	Unimportant	Should not be included
*provide specific examples of the person's behaviours and their impact on the person, as well as the person's family and friends.	○	○	○	○	○
*tell the person what they will do if they refuse to accept treatment.	○	○	○	○	○
*try to get the person to make an on-the-spot decision.	○	○	○	○	○
*give the person a few days to weigh their options for seeking professional help.	○	○	○	○	○

Q299. The first aider should be aware that an intervention may not be successful.

- Essential
- Important
- Don't know/Depends
- Unimportant
- Should not be included

Q300. The first aider should be aware the person may respond negatively to the intervention.

- Essential
- Important
- Don't know/Depends
- Unimportant
- Should not be included

Q301. Do you have any comments on the above statements? Is there anything you would like to add? Please write your suggestions in the box provided.

Crisis situations

Q302. **Crisis situations**

This section asks you what the first aider should do in a crisis situation.

Please rate how important (from 'essential' to 'should not be included') you think it is that each statement be included in the guidelines.

Please also keep the **definitions** in mind when rating the statements.

Please note, within this section there are some statements that only some panel members will have the expertise to answer. If you do not have the expertise to answer a question, please select the option "I do not have the expertise to answer this question."

Q303. **Recognising a crisis**

Q304. The first aider should be able to recognise the signs of intoxication.

- Essential
- Important
- Don't know/Depends
- Unimportant
- Should not be included
- I do not have the expertise to answer this question

Q305. The first aider should be able to recognise the signs of a substance-related medical emergency.

- Essential
- Important
- Don't know/Depends
- Unimportant
- Should not be included
- I do not have the expertise to answer this question

Q306. The first aider should be aware that symptoms of other medical conditions can mimic the symptoms of substance affected states.

- Essential
- Important
- Don't know/Depends
- Unimportant
- Should not be included
- I do not have the expertise to answer this question

Q307. The first aider should be aware that the effects of substances vary from person to person depending on a number of factors, e.g. speed of consumption, whether they have eaten anything, body size and age.

- Essential
- Important
- Don't know/Depends

- Unimportant
- Should not be included
- I do not have the expertise to answer this question

Q308. The first aider should be aware that the amount of substances that causes poisoning or an overdose is different for every person.

- Essential
- Important
- Don't know/Depends
- Unimportant
- Should not be included
- I do not have the expertise to answer this question

Q309. The first aider should be able to recognise when the physical condition of a person who has been using substances is getting worse (e.g. they become unconscious, start vomiting uncontrollably), and call emergency services.

- Essential
- Important
- Don't know/Depends
- Unimportant
- Should not be included
- I do not have the expertise to answer this question

Q310. **Intoxication- how to communicate**

Q311. If the person is intoxicated, the first aider should:

	Essential	Important	Don't know/Depends	Unimportant	Should not be included	I do not have the expertise to answer this question
*stay calm.	O	O	O	O	O	O
*talk with them in a calm, reassuring and respectful manner.	O	O	O	O	O	O
*talk with them using simple, clear language and repeating when necessary.	O	O	O	O	O	O

Q312. The first aider should be aware that the intoxicated person may overreact to negative words; therefore, the first aider should use positive words (such as ‘stay calm’) instead of negative words (such as ‘don’t fight’).

- O Essential
- O Important
- O Don't know/Depends
- O Unimportant
- O Should not be included
- O I do not have the expertise to answer this question

Q313. The first aider should not:

	Essential	Important	Don't know/Depends	Unimportant	Should not be included	I do not have the expertise to answer this question
*laugh at, make fun of, or provoke the intoxicated person.	O	O	O	O	O	O
*speak in an angry manner.	O	O	O	O	O	O

	Essential	Important	Don't know/Depends	Unimportant	Should not be included	I do not have the expertise to answer this question
*attempt to engage the intoxicated person in a serious conversation about their substance use.	○	○	○	○	○	○

Q314. **Monitor for danger**

Q315. The first aider should assess the situation for potential dangers and ensure that the intoxicated person, themselves and others are safe.

- ○ Essential
- ○ Important
- ○ Don't know/Depends
- ○ Unimportant
- ○ Should not be included
- ○ I do not have the expertise to answer this question

Q316. The first aider should monitor the intoxicated person and their environment to prevent injury.

- ○ Essential
- ○ Important
- ○ Don't know/Depends
- ○ Unimportant
- ○ Should not be included
- ○ I do not have the expertise to answer this question

Q317. The first aider should ask the intoxicated person what medications and substances

they have taken, in case the person's condition deteriorates into a medical emergency.

- Essential
- Important
- Don't know/Depends
- Unimportant
- Should not be included
- I do not have the expertise to answer this question

Q318. The first aider should be aware that substance use can mask pain from injuries.

- Essential
- Important
- Don't know/Depends
- Unimportant
- Should not be included
- I do not have the expertise to answer this question

Q319. If the person is affected by substances, the first aider should try to maintain a safe distance and try to appear non-threatening.

- Essential
- Important
- Don't know/Depends
- Unimportant
- Should not be included
- I do not have the expertise to answer this question

Q320. The first aider should watch the intoxicated person for signs of increasing aggression.

- Essential
- Important
- Don't know/Depends
- Unimportant

- Should not be included
- I do not have the expertise to answer this question

Q321. If the person is experiencing a crisis, the first aider should discourage the person from taking any more substances.

- Essential
- Important
- Don't know/Depends
- Unimportant
- Should not be included
- I do not have the expertise to answer this question

Q322. **Keep the person safe**

Q323. The first aider should stay with the intoxicated person or ensure they are not left alone.

- Essential
- Important
- Don't know/Depends
- Unimportant
- Should not be included
- I do not have the expertise to answer this question

Q324. If the person is heavily intoxicated and the first aider feels uncomfortable monitoring them, the first aider should take the person to a sobering up shelter or drug and alcohol service, if available.

- Essential
- Important
- Don't know/Depends
- Unimportant
- Should not be included

- I do not have the expertise to answer this question

Q325. If the first aider has been using substances, they should enlist the help of someone who is sober to assist the person.

- Essential
- Important
- Don't know/Depends
- Unimportant
- Should not be included
- I do not have the expertise to answer this question

Q326. The first aider should prevent the intoxicated person from walking off alone, but only if it is safe for the first aider to do so.

- Essential
- Important
- Don't know/Depends
- Unimportant
- Should not be included
- I do not have the expertise to answer this question

Q327. The first aider should be aware that when intoxicated the person may engage in a wide range of risky activities, such as having unprotected sex, arguments or fights, or driving a car.

- Essential
- Important
- Don't know/Depends
- Unimportant
- Should not be included
- I do not have the expertise to answer this question

Q328. The first aider should try to prevent the person from taking part in risky or unsafe activities.

- Essential
- Important
- Don't know/Depends
- Unimportant
- Should not be included
- I do not have the expertise to answer this question

Q329. The first aider should organise a safe mode of transport for the intoxicated person.

- Essential
- Important
- Don't know/Depends
- Unimportant
- Should not be included
- I do not have the expertise to answer this question

Q330. The first aider should be aware that the intoxicated person may be at risk of being a victim of crime.

- Essential
- Important
- Don't know/Depends
- Unimportant
- Should not be included
- I do not have the expertise to answer this question

Q331. The first aider should advise the intoxicated person not to drive the next day or engage in other tasks that involve risk of injury.

- Essential
- Important

- Don't know/Depends
- Unimportant
- Should not be included
- I do not have the expertise to answer this question

Q332. **Awareness on the impact of intoxication**

Q333. The first aider should be aware that illicit substances can have unpredictable effects as they are not manufactured in a controlled way.

- Essential
- Important
- Don't know/Depends
- Unimportant
- Should not be included
- I do not have the expertise to answer this question

Q334. The first aider should be aware that it is often difficult to differentiate between the effects of different substances on behaviour.

- Essential
- Important
- Don't know/Depends
- Unimportant
- Should not be included
- I do not have the expertise to answer this question

Q335. The first aider should be aware that only time will reverse the effects of alcohol intoxication.

- Essential
- Important
- Don't know/Depends

- Unimportant
- Should not be included
- I do not have the expertise to answer this question

Q336. The first aider should be aware that drinking black coffee, sleeping, walking and cold showers will not reverse the effects of intoxication.

- Essential
- Important
- Don't know/Depends
- Unimportant
- Should not be included
- I do not have the expertise to answer this question

Q337. **Medical emergencies**

Q338. The first aider should know the local emergency services physical first aid guidelines for intoxication and overdose.^

- Essential
- Important
- Don't know/Depends
- Unimportant
- Should not be included
- I do not have the expertise to answer this question

Q339. ^ Physical first aid for Alcohol, Stimulants, Opioids, Depressants

Q340. The first aider should be able to recognise the symptoms of substance use withdrawal.^

- Essential

- Important
- Don't know/Depends
- Unimportant
- Should not be included
- I do not have the expertise to answer this question

Q341. ^ **Symptoms of substance use withdrawal:**
Insomnia, irritability, changing moods, depression, anxiety, aches and pains, cravings, fatigue, hallucinations, nausea snd seizures. The person may be hot and cold, have goosebumps, or have a runny nose as if they have a cold.

Severe withdrawal symptoms: paranoia, confusion, tremors and disorientation.

Adapted from: https://www.healthdirect.gov.au/addiction-withdrawal-symptoms

Q342. The first aider should seek emergency help if the person displays symptoms of severe withdrawal.^

- Essential
- Important
- Don't know/Depends
- Unimportant
- Should not be included
- I do not have the expertise to answer this question

Q343. **^ Symptoms of severe substance use withdrawal:** paranoia, confusion, tremors and disorientation.

Adapted from: https://www.healthdirect.gov.au/addiction-withdrawal-symptoms

Q344. **What to do if the person is aggressive**

Q345. The first aider should not stay with the person if the first aider's own safety is at risk.

- Essential
- Important
- Don't know/Depends
- Unimportant
- Should not be included
- I do not have the expertise to answer this question

Q346. If the first aider is feeling unsafe, they should seek other supports, e.g. involve the person's family members or friends.

- Essential
- Important
- Don't know/Depends
- Unimportant
- Should not be included
- I do not have the expertise to answer this question

Q347. If the person becomes aggressive, the first aider should:

	Essential	Important	Don't know/Depends	Unimportant	Should not be included	I do not have the expertise to answer this question
*assess the risks to themselves, the person and others, before trying to assist.	○	○	○	○	○	○
*prioritise their own safety.	○	○	○	○	○	○
*keep a safe distance from the person.	○	○	○	○	○	○
*if inside, try to keep the exits clear so	○	○	○	○	○	○

	Essential	Important	Don't know/Depends	Unimportant	Should not be included	I do not have the expertise to answer this question
that the person does not feel penned in and the first aider and others can get away easily if needed.						
*consider taking a break from the conversation to allow the intoxicated person a chance to calm down.	O	O	O	O	O	O
*talk to the person in a calm, non-confrontational manner.	O	O	O	O	O	O
*try to remain as calm as possible.	O	O	O	O	O	O
*speak slowly and confidently.	O	O	O	O	O	O
*repeat things if necessary.	O	O	O	O	O	O
*avoid asking too many questions, as it could make the person more angry.	O	O	O	O	O	O
*speak with a gentle, caring tone of voice.	O	O	O	O	O	O
*try not to provoke the person.	O	O	O	O	O	O
*avoid arguing with the intoxicated person.	O	O	O	O	O	O
*avoid displaying nervous behaviour, e.g. shuffling their feet, fidgeting or making abrupt movements.	O	O	O	O	O	O

	Essential	Important	Don't know/Depends	Unimportant	Should not be included	I do not have the expertise to answer this question
*tell the person what they are going to do before doing it, e.g. calling for help or asking others to leave.	○	○	○	○	○	○
*try to provide the person with a quiet environment away from noise and other distractions.	○	○	○	○	○	○
*tell the person that no-one will hurt them.	○	○	○	○	○	○
*reassure the person that they are there to help.	○	○	○	○	○	○
*acknowledge the person's agitation, e.g. "I can see that you are upset."	○	○	○	○	○	○
*ask the person what they want and then repeat what they requested.	○	○	○	○	○	○
*move slowly.	○	○	○	○	○	○
*try not to make too much eye contact	○	○	○	○	○	○
*make sure the area is safe as possible, e.g. removing dangerous objects.	○	○	○	○	○	○
*try and use the person's name, e.g. "Jason, can you tell me what is going on for you?"	○	○	○	○	○	○

Q348. The first aider should continually reassess if what they are doing is helping; if not, then they should try something different.

- Essential
- Important
- Don't know/Depends
- Unimportant
- Should not be included
- I do not have the expertise to answer this question

Q349. The first aider should discourage the person from using any more substances in the short term.

- Essential
- Important
- Don't know/Depends
- Unimportant
- Should not be included
- I do not have the expertise to answer this question

Q350. The first aider should get help if the person is going to hurt themselves or someone else.

- Essential
- Important
- Don't know/Depends
- Unimportant
- Should not be included
- I do not have the expertise to answer this question

Q351. If the person becomes aggressive, the first aider should only call the police if all other avenues of de-escalation have been exhausted.

- Essential

- Important
- Don't know/Depends
- Unimportant
- Should not be included
- I do not have the expertise to answer this question

Q352. The first aider should only call the police if all other avenues of de-escalation have been exhausted and the person is at risk of harming themselves or others.

- Essential
- Important
- Don't know/Depends
- Unimportant
- Should not be included
- I do not have the expertise to answer this question

Q353. If violence occurs, the first aider should seek the appropriate emergency assistance.

- Essential
- Important
- Don't know/Depends
- Unimportant
- Should not be included
- I do not have the expertise to answer this question

Q354. If violence has been prevented, the first aider should avoid discussing the incident unless the affected person brings it up.

- Essential
- Important
- Don't know/Depends
- Unimportant
- Should not be included
- I do not have the expertise to answer this question

Q355. **Adverse psychological reactions**

Q356. The first aider should be aware it can be difficult to differentiate between the symptoms of mental illness and substance-affected behaviour.

- Essential
- Important
- Don't know/Depends
- Unimportant
- Should not be included
- I do not have the expertise to answer this question

Q357. The first aider should be able to recognise adverse psychological reactions to substances.

- Essential
- Important
- Don't know/Depends
- Unimportant
- Should not be included
- I do not have the expertise to answer this question

Q358. The first aider should monitor the person in case their psychological state deteriorates.

- Essential
- Important
- Don't know/Depends
- Unimportant
- Should not be included
- I do not have the expertise to answer this question

Q359. The first aider should be aware that, when intoxicated, the person may be at higher risk of attempting suicide.

- Essential
- Important
- Don't know/Depends
- Unimportant
- Should not be included
- I do not have the expertise to answer this question

Q360. The first aider should monitor for signs that the intoxicated person is suicidal.

- Essential
- Important
- Don't know/Depends
- Unimportant
- Should not be included
- I do not have the expertise to answer this question

Q361. The first aider should be aware of the Mental Health First Aid Guidelines for how to assist someone with suicidal thoughts or behaviours.

- Essential
- Important
- Don't know/Depends
- Unimportant
- Should not be included
- I do not have the expertise to answer this question

Q362. If the affected person is anxious and panicky, the first aider should take them to a quiet environment away from crowds, loud noise and bright lights.

- Essential
- Important

- Don't know/Depends
- Unimportant
- Should not be included
- I do not have the expertise to answer this question

Q363. The first aider should be aware of the Mental Health First Aid Guidelines for how to assist someone experiencing a panic attack.

- Essential
- Important
- Don't know/Depends
- Unimportant
- Should not be included
- I do not have the expertise to answer this question

Q364. The first aider should know how to help the affected person if they are experiencing psychosis or psychotic symptoms.

- Essential
- Important
- Don't know/Depends
- Unimportant
- Should not be included
- I do not have the expertise to answer this question

Q365. The first aider should be aware that psychotic symptoms could be substance-related.

- Essential
- Important
- Don't know/Depends
- Unimportant
- Should not be included
- I do not have the expertise to answer this question

Q366. The first aider should be aware of the **Mental Health First Aid Guidelines** for how to assist someone experiencing psychosis or psychotic symptoms.

- Essential
- Important
- Don't know/Depends
- Unimportant
- Should not be included
- I do not have the expertise to answer this question

Q367. Do you have any comments on the above statements? Is there anything you would like to add? Please write your suggestions in the box provided.

Adolescents

Q368. **Adolescents**

This section asks you what the first aider should consider if an adolescent is experiencing substance use problems.

Please rate how important (from 'essential' to 'should not be included') you think it is that each statement be included in the guidelines.

Please also keep the **definitions** in mind when rating the statements.

Q369. The first aider should be aware of the impact of substances on the development of the adolescent.

- Essential
- Important
- Don't know/Depends

- ○ Unimportant
- ○ Should not be included

Q370. Do you have any comments on the above statement? Is there anything you would like to add? Please write your suggestions in the box provided.

Powered by Qualtrics

Round 2 Survey

R2 Instructions

Round 2 survey: Updating the Mental Health First Aid guidelines for problem substance use

Your participation

Thank you for your participation in this project so far.

As advised, participation in this project involves completing three rounds of online surveys. Thank you for completing the first survey earlier this year. It is now time to complete the second (shorter) survey.

Purpose of the research

The aim of this current research project is to update the mental health first aid guidelines for how a member of the public should give assistance to a person who is experiencing problem alcohol or other drug use.

Instructions

Your task is to complete the survey by rating each statement according to how important you believe it is for inclusion in the guidelines for providing mental health first aid for someone who is experiencing problem alcohol or other drug use. This involves re-rating some items from Round 1 and rating some new items.

Please keep in mind that the guidelines will be used by the general public. The statements need to be rated according to their importance for someone WITHOUT a counselling or clinical background.

This questionnaire should take approximately 30 - 60 minutes to complete. You can complete the survey in two or more sittings. Your answers are saved when you click 'Next' at the bottom of the page. This marks your page and you can begin again at a later date on the next page. Please be aware that once you have logged on and started responding you must complete the questionnaire on the same computer.

How this questionnaire was developed

The statements in this questionnaire were derived from the results of the Round 1 survey. You will note that each statement is marked as either a NEW or RERATE item:

NEW ITEMS: These are new items that were derived from the comments provided in the first survey.

RE-RATE ITEMS: These are items from the first survey that were neither endorsed nor rejected by the expert groups. An item is rerated when 70% - 79% of panel members from the expert group rated it as essential or important.

It is important to remember that we do not necessarily agree with these statements, and some may seem contradictory or controversial. The items have been included because they reflect a wide range of people's beliefs about intervention and care. Your role is to provide use with your opinion to inform the development of a set of guidelines that reflect current expert opinion.

As advised, you may wish to use information from your Round 1 Report to inform how you respond to re-rate items in this round. Please note, the item numbers in the Round 1 Report correspond to those listed in the Round 2 survey in parentheses after each re-rate item, for example - (RE-RATE Item #14).

Consent to participate

It is important for you to know that participation in this study is completely voluntary. You are not under any obligation to participate and can withdraw at any time.

We would like to thank you for your time and effort, and encourage you to provide us with feedback on this process.

Who can I contact if I have any concerns about the project?

This reseach project has been approved by the Human Research Ethics Committee of The University of Melbourne. If you have any concerns or complaints about the conduct of this research project which you do not wish to discuss with the research team, you should contact the Manager, Human Research Ethics, Research Ethicxs and Integrity, University of Melbourne, VIC 3010 on Tel: +61 3 8344 2073 or Email: HumanEthics-complaints@unimelb.edu.au. All complaints will be treated confidentially. In any correspondence, please provide the ethics ID number. **The ethics ID number of the research project is 1851765.**

For more information

You received a Plain Language Statement when you expressed interest in this project (also available here). Please refer to this for more details about this study. You may also contact Judith Wright via email for further information: wright.j@unimelb.edu.au.

Definitions

Definitions used in this survey

Mental health first aid is the help provided to a person who is developing a mental health problem, experiencing a worsening of an existing mental health problem or in a mental health

crisis. The first aid is given until appropriate professional help is received or the crisis resolves.

The person: someone who is experiencing substance use problems.

The first aider: a concerned family member, friend, work colleague or community member, who provides help to a person experiencing substance use problems.

Professional: a broad range of relevantly trained health professionals. This could include a mental health professional, GP/family doctor, hospital emergency staff, ambulance officer or paramedic.

Emergency services: services that respond to and deal with emergencies when they occur, e.g. emergency medical services (ambulance) or law enforcement (the police).

Substances: psychoactive compounds, including alcohol or other drugs. Substances may be legal or illegal.

Substance use problems/Problem substance use: a pattern of harmful use of substances that has the potential to negatively impact a person's physical and mental health, relationships, employment, finances, and the safety of themselves and others. They may or may not have a substance use disorder.

Substance dependence: refers to heavy, regular substance use that results in the person having difficulties controlling the amount they use and experiencing symptoms (such as anxiety) when they stop using or use less than usual. Substance dependence occurs when someone feels they need to use substances in order to get through their day or week.

Lapse: a short (or temporary) departure from a person's substance use goals followed by a return to their goals.

Relapse: a return to previous levels of substance use after a period of sustained reduction or avoidance of substances.

Withdrawal: refers to a physical condition that occurs when a person, who has been using substances heavily or for extended periods of time, stops using or uses substantially less than usual. Symptoms of withdrawal include severe anxiety, headaches and tremors or shakes. Withdrawal can be a life-threatening condition.

Overdose: is when too much of a substance/s is taken causing harmful effects on the body. Overdoses may be accidental or intentional and can involve substances that are prescription, over-the-counter, legal, or illegal. A person can overdose on many substances, including alcohol (alcohol poisoning), stimulants, pain medication or a mix of substances. An overdose can lead to

serious medical complications, including death.

Overview of the questionnaire

Section 1: What should the first aider know about substance use problems?
Section 2: When does the person need help?
Section 3: Approaching the person
Section 4: Providing information
Section 5: Providing support
Section 6: Supporting someone with a history of substance use problems
Section 7: Professional help
Section 8: Interventions
Section 9: Crisis situations
Section 10: Adolescents

Information about you

Information about you

Q1. What is your name?

(This allows us to determine who has completed the Round 1 survey and is therefore eligible to participate in Round 2. Your name will be deleted from your data when the project is complete).

Section 1: What should the first aider know about substance us problems?

Section 1: What should the first aider know about substance use problems?

This section asks you what information the first aider should know in order to assist the person experiencing substance use problems.

Please rate how important (from ‘essential’ to ‘should not be included’) you think it is that each statement be included in the guidelines.

Please also keep the definitions in mind when rating the statements

1.1 What are substance use problems?

Q1. The first aider should be aware that persons with problem substance use may be very sensitive to stereotyping and the judgements of others. (NEW)

- Essential
- Important
- Don't know/Depends
- Unimportant
- Should not be included

Q2. The first aider should be aware of both the social and individual risk factors for problem substance use. (NEW)

- Essential
- Important
- Don't know/Depends
- Unimportant
- Should not be included

Q3. The first aider should be aware that traumatic experiences may have contributed to the person's substance use problems. (NEW)

- Essential
- Important
- Don't know/Depends
- Unimportant
- Should not be included

1.3 Co-occurring mental health problems

Q4. The first aider should be aware of the high co-occurrence between problem substance use and other mental health problems. (NEW)

- Essential
- Important
- Don't know/Depends
- Unimportant
- Should not be included

Section 2: When does the person need help?

Section 2: When does the person need help?

This section asks you what the first aider should know in order to recognise when the person needs help.

Please rate how important (from ‘essential’ to ‘should not be included’) you think it is that each statement be included in the guidelines.

Please also keep the definitions in mind when rating the statements

2.2 Prioritising others' safety

Q5. If the first aider becomes aware that the person’s substance use is placing the safety of others (e.g. partner or family members) at risk, the first aider's priority should be to keep these people safe. (RE-RATE: Item #13)

- Essential
- Important
- Don't know/Depends
- Unimportant
- Should not be included

Q6. If the first aider becomes aware that the person’s substance use is placing the safety of others (e.g. partner or family members) at risk, the first aider's priority should be to keep these people safe if practical and safe to do so. (NEW)

- Essential
- Important
- Don't know/Depends
- Unimportant
- Should not be included

Q7. If the first aider becomes aware that the person's substance use is placing the safety of other adults (e.g. partner or adult family members) at risk, the first aider should offer them information on resources available to them. (NEW)

- Essential
- Important
- Don't know/Depends
- Unimportant
- Should not be included

2.3 Warning signs

Q8. The first aider should be aware of common physical health signs and symptoms of problem substance use. (NEW)

- Essential
- Important
- Don't know/Depends
- Unimportant
- Should not be included

Q9. The first aider should know the following warning signs for recognising substance use problems:

	Essential	Important	Don't know/Depends	Unimportant	Should not be included
The person plays down how much they use substances. (RE-RATE: Item #15)	○	○	○	○	○
The person behaves irrationally. (RE-RATE: Item #18)	○	○	○	○	○

	Essential	Important	Don't know/Depends	Unimportant	Should not be included
The person behaves in uncharacteristic ways. (NEW)	○	○	○	○	○
The person has become isolated from both family and friends. (RE-RATE: Item #27)	○	○	○	○	○
The person spends increasingly less time with family and friends. (NEW)	○	○	○	○	○
The person is neglecting their responsibilities. (RE-RATE: Item #28)	○	○	○	○	○
The person is not meeting home, social or work obligations and responsibilities. (NEW)	○	○	○	○	○
The person uses increasingly larger amounts of substances and more often over time. (NEW)	○	○	○	○	○
The person does not recall events. (RE-RATE: Item #36)	○	○	○	○	○
The person uses the substance alone, in the morning or for hours on end. (RE-RATE: Item #37)	○	○	○	○	○

Section 3: Approaching the person

Section 3: Approaching the person

This section asks you what the first aider should know and do when approaching the person.

Please rate how important (from ‘essential’ to ‘should not be included’) you think it is that each statement be included in the guidelines.

Please also keep the definitions in mind when rating the statements

3.1 Considerations before making an approach

Q10. The first aider should not assume the person is experiencing a substance use problem. (NEW)

- Essential
- Important
- Don't know/Depends
- Unimportant
- Should not be included

Q11. The first aider should be aware the person may feel more comfortable seeking support from someone with lived experience of substance use problems who they can relate to. (NEW)

- Essential
- Important
- Don't know/Depends
- Unimportant
- Should not be included

Q12. The first aider should be aware that any positive change towards reducing substance use is beneficial. (NEW)

- Essential
- Important
- Don't know/Depends
- Unimportant
- Should not be included

3.2 Support for providing first aid

Q13. The first aider should be aware that they can contact a helpline for support in assisting someone with substance use problems. (NEW)

- Essential
- Important
- Don't know/Depends
- Unimportant
- Should not be included

Q14. The first aider should let the person know they can contact a helpline for support when they are ready for change or experiencing a craving or distress. (NEW)

- Essential
- Important
- Don't know/Depends
- Unimportant
- Should not be included

Q15. The first aider should consider contacting someone with lived experience of substance use problems before contacting a health professional to determine how best to approach the person about their concerns. (NEW)

- Essential
- Important
- Don't know/Depends
- Unimportant
- Should not be included

Q16. The first aider should always seek professional advice when considering how best to approach the person. (NEW)

- Essential
- Important
- Don't know/Depends
- Unimportant
- Should not be included

3.3 Specific planning

Q17. The first aider should reflect on the person's situation, organise their own thoughts and decide what they want to say to the person. (RE-RATE: Item #50)

- Essential
- Important
- Don't know/Depends

- Unimportant
- Should not be included

3.4 Be aware of potential outcomes

Q18. The first aider should try not to take personally things the person has said or done when they are affected by substances. (NEW)

- Essential
- Important
- Don't know/Depends
- Unimportant
- Should not be included

Section 3: Approaching the person (continued)

3.5 When to talk

Q19. The first aider should speak to the person when they are not affected by substances if possible. (NEW)

- Essential
- Important
- Don't know/Depends
- Unimportant
- Should not be included

Q20. If the first aider is only able to speak to the person while they are affected by substances, the first aider should try to approach them when they are in a lucid, co-operative state. (NEW)

- Essential
- Important
- Don't know/Depends
- Unimportant
- Should not be included

3.6 Interpersonal skills

Q21. The first aider should use open questions that encourage the person to think about their substance use, e.g. "What do you think about your alcohol use? How do you think you can change it?". (RE-RATE: Item #77)

- Essential
- Important
- Don't know/Depends
- Unimportant
- Should not be included

Q22. The first aider should stick to the point (i.e. focus on the person’s substance use) and not get drawn into arguments about other issues. (RE-RATE: Item #78)

- Essential
- Important
- Don't know/Depends
- Unimportant
- Should not be included

Q23. The first aider should try to keep the focus of the conversation on the person's substance use if other issues are raised. (NEW)

- Essential
- Important
- Don't know/Depends
- Unimportant
- Should not be included

3.7 What to say and do

Q24. The first aider should ask the person about the reasons for their substance use. (NEW)

- Essential

- Important
- Don't know/Depends
- Unimportant
- Should not be included

Q25. The first aider should tell the person that they are capable of change if that is what they want. (NEW)

- Essential
- Important
- Don't know/Depends
- Unimportant
- Should not be included

Q26. The first aider should discuss with the person that stopping or reducing substance use is hard, it may be painful, and it takes time. (RE-RATE: Item #97)

- Essential
- Important
- Don't know/Depends
- Unimportant
- Should not be included

Q27. The first aider should advise the person that alcohol may interact with other drugs (illicit or prescribed) in an unpredictable way which may lead to a medical emergency. (RE-RATE: Item #98)

- Essential
- Important
- Don't know/Depends
- Unimportant
- Should not be included

Section 3: Approaching the person (continued)

3.8 What to avoid

Q28. The first aider should try to avoid self-disclosure that brings the focus away from the person. (NEW)

- Essential
- Important
- Don't know/Depends
- Unimportant
- Should not be included

Q29. If the first aider has their own lived experience with substance use, they should not discuss it with the person. (NEW)

- Essential
- Important
- Don't know/Depends
- Unimportant
- Should not be included

3.9 What to do if the person does not believe they have a problem

Q30. If the person does not believe they have a problem, the first aider should continue to provide social support. (NEW)

- Essential
- Important
- Don't know/Depends
- Unimportant
- Should not be included

Q31. If the person does not believe they have a problem, the first aider should encourage the person to reduce harms related to their substance use. (NEW)

- Essential
- Important

- Don't know/Depends
- Unimportant
- Should not be included

3.11 Encouraging change

Q32. The first aider should provide the person with some information about how to cut down on their substance use. (RE-RATE: Item #123)

- Essential
- Important
- Don't know/Depends
- Unimportant
- Should not be included

Q33. If the person is interested in cutting down on their substance use, the first aider should provide the person with some information about how to do this. (NEW)

- Essential
- Important
- Don't know/Depends
- Unimportant
- Should not be included

Q34. If the first aider provides any information to the person, they should make sure it is from a reputable source. (NEW)

- Essential
- Important
- Don't know/Depends
- Unimportant
- Should not be included

Section 4: Providing Information

Section 4: Providing information

This section asks you what information the first aider should provide to the person.

Please rate how important (from 'essential' to 'should not be included') you think it is that each statement be included in the guidelines.

Please also keep the definitions in mind when rating the statements.

4.1 What information should the first aider provide?

Q35. The first aider should be aware that ceasing to use a substance without medical advice can be dangerous. (NEW)

- Essential
- Important
- Don't know/Depends
- Unimportant
- Should not be included

Q36. If the person is open to receiving information, the first aider should:

	Essential	Important	Don't know/Depends	Unimportant	Sh no incl
Offer the person some information about substance use problems. (NEW)	○	○	○	○	○
Provide some basic facts about substance use problems, e.g. how common they are, the associated risks, available treatments. (NEW)	○	○	○	○	○
Help the person get some information on how to change their problem substance use. (NEW)	○	○	○	○	○
Discuss with the person some risks associated with problem substance use. (NEW)	○	○	○	○	○
Offer to help the person get information on online screening questionnaires. (NEW)	○	○	○	○	○

Q37. The first aider should offer to help the person get information on a range of local treatment options and allow the person to decide which would be most appropriate or useful for them. (RE-RATE: Item #133)

- Essential
- Important
- Don't know/Depends
- Unimportant
- Should not be included

Q38. The first aider should be prepared to offer information for group support or self-help strategies if the person needs them. (NEW)

- Essential
- Important
- Don't know/Depends
- Unimportant
- Should not be included

4.2 Harm reduction

Q39. The first aider should have an understanding of harm reduction. (NEW)

- Essential
- Important
- Don't know/Depends
- Unimportant
- Should not be included

Q40. If the person wants help to change their substance use, the first aider should ask what type of help and support they would find most helpful. (NEW)

- Essential
- Important
- Don't know/Depends
- Unimportant

- Should not be included

Q41. The first aider should provide the person with information about harm reduction strategies. (RE-RATE: Item #142)

- Essential
- Important
- Don't know/Depends
- Unimportant
- Should not be included

Q42. The first aider should offer the person information about harm reduction strategies. (NEW)

- Essential
- Important
- Don't know/Depends
- Unimportant
- Should not be included

Q43. The first aider should encourage the person to avoid engaging in activities that may pose a risk to others while using substances, e.g. driving, working, and looking after children. (NEW)

- Essential
- Important
- Don't know/Depends
- Unimportant
- Should not be included

Q44. If the person using opioids, the first aider should discuss with the person the benefits of having naloxone accessible. (NEW)

- Essential
- Important
- Don't know/Depends
- Unimportant
- Should not be included

Q45. If the person's substance use problems include alcohol the first aider should ask the person if they would like some tips on reducing their health risks when drinking. (RE-RATE: Item #144)

PRACTICAL TIPS FOR REDUCING HEALTH RISKS WHEN DRINKING

- Know how much alcohol is in a standard drink
- Know the number of standard drinks in each beverage (the number of standard drinks is often listed on the beverage’s packaging)
- Keep count of the number of standard drinks consumed
- Do not let people top up your glass before it is finished, so as not to lose track of how much alcohol has been consumed
- Eat while drinking
- Drink plenty of water when drinking alcohol to prevent dehydration
- Drink beverages with lower alcohol content (e.g. low-alcohol beer instead of full strength beer)
- Switch to non-alcoholic drinks when starting to feel the effects of alcohol
- Avoid keeping up with friends drink for drink
- Avoid drinking competitions and drinking games
- Drink slowly, for example, by taking sips instead of gulps and putting the drink down between sips
- Only have one drink at a time
- Spend time on activities that don’t involve drinking
- Drink alcohol as part of another activity instead of making it the main activity
- Identify situations where drinking is likely and avoid them if possible

- ○ Essential
- ○ Important
- ○ Don't know/Depends
- ○ Unimportant
- ○ Should not be included

Q46. If the person is interested in harm reduction strategies, the first aider should ask if they can assist with this in any way. (NEW)

- ○ Essential
- ○ Important
- ○ Don't know/Depends
- ○ Unimportant

- Should not be included

Section 5: Providing support

Section 5: Supporting the person

This section asks you how the first aider should support the person.

Please rate how important (from 'essential' to 'should not be included') you think it is that each statement be included in the guidelines.

Please also keep the definitions in mind when rating the statements.

5.1 How to support the person if they are unwilling to change

Q47. If the person is unwilling to change their substance use, the first aider should not:

	Essential	Important	Don't know/Depends	Unimportant	Sho not inclu
Take responsibility for the person's recovery journey. (NEW)	○	○	○	○	○
Get involved with helping the person obtain substances, e.g. driving the person to meet a dealer. (RE-RATE: Item #163)	○	○	○	○	○

5.2 How to support the person if they are willing to change

Q48. If the person wants to stop or reduce their substance use, the first aider should encourage the person to seek professional advice before they change their substance use behaviour. (RE-RATE: Item #181)

- Essential
- Important
- Don't know/Depends
- Unimportant
- Should not be included

Q49. If the person has been drinking for long periods, the first aider should tell them not to suddenly stop drinking without first seeking medical help to avoid the possibility of withdrawal which can be fatal. (NEW)

- Essential
- Important
- Don't know/Depends
- Unimportant
- Should not be included

Q50. The first aider should warn the person that not all family and friends will be supportive of their efforts to change their substance use. (RE-RATE: Item #190)

- Essential
- Important
- Don't know/Depends
- Unimportant
- Should not be included

Q51. The first aider should suggest the person connect with other people's recovery stories, e.g. in-person support groups, consumer events, or online. (NEW)

- Essential
- Important
- Don't know/Depends
- Unimportant
- Should not be included

Q52. The first aider should encourage the person to talk to a friend or someone they trust about their problems, not just their substance use. (RE-RATE: Item #192)

- Essential
- Important
- Don't know/Depends
- Unimportant

- Should not be included

Q53. The first aider should encourage the person to spend time with supportive family or friends who don't use substances. (NEW)

- Essential
- Important
- Don't know/Depends
- Unimportant
- Should not be included

5.3 Encouraging other supports

Q54. The first aider should discuss with the person realistic lifestyle changes they could adopt without overwhelming them with options. (NEW)

- Essential
- Important
- Don't know/Depends
- Unimportant
- Should not be included

Q55. The first aider should suggest to the person that they reconnect with past hobbies or interests. (NEW)

- Essential
- Important
- Don't know/Depends
- Unimportant
- Should not be included

Section 5: Supporting the person (continued)

5.4 Managing social pressure

Q56. The first aider should recommend that the person have an "exit plan" they can use in social situations that may tempt them to use substances. (NEW)

- Essential
- Important
- Don't know/Depends
- Unimportant
- Should not be included

Q57. The first aider should have an understanding of the different substances used in the person's community. (RE-RATE: Item #200)

- Essential
- Important
- Don't know/Depends
- Unimportant
- Should not be included

Q58. The first aider should reassure the person that they do not need to explain to others why they are not using substances. (RE-RATE: Item #204)

- Essential
- Important
- Don't know/Depends
- Unimportant
- Should not be included

Q59. The first aider should suggest the person try to stay away from people who pressure them to use substances. (RE-RATE: Item #205)

- Essential
- Important
- Don't know/Depends
- Unimportant
- Should not be included

Q60. The first aider should tell the person that the people who care about their well-being will accept their decision not to use. (NEW)

- Essential
- Important
- Don't know/Depends
- Unimportant
- Should not be included

5.5 Providing support with boundaries

Q61. The first aider should not make excuses or cover up the behaviour of the person to protect them from the consequences of their substance use. (RE-RATE: Item #210)

- Essential
- Important
- Don't know/Depends
- Unimportant
- Should not be included

5.6 Healthy lifestyle

Q62. The first aider should encourage the person to eat healthily so their body can be strong while they are trying to change their problem substance use. (RE-RATE: Item #214)

- Essential
- Important
- Don't know/Depends
- Unimportant
- Should not be included

Q63. The first aider should encourage the person to try and get a good amount of sleep so their body can work to repair itself while they are trying to change their problem substance use. (RE-RATE: Item #215)

- Essential

- Important
- Don't know/Depends
- Unimportant
- Should not be included

Q64. The first aider should encourage the person to find healthy ways to feel good instead of using substances. (RE-RATE: Item #216)

- Essential
- Important
- Don't know/Depends
- Unimportant
- Should not be included

5.7 Disclosure

Q65. The first aider should not disclose the person's problem substance use to another adult unless the person is a child or adolescent. (NEW)

- Essential
- Important
- Don't know/Depends
- Unimportant
- Should not be included

Q66. The first aider should not disclose the person’s problem substance use to a professional unless the person is a child or young person. (RE-RATE: Item #224)

- Essential
- Important
- Don't know/Depends
- Unimportant
- Should not be included

Q67. The first aider should not disclose the person's problem substance use to a professional unless the person is a child or adolescent up to 18 years. (NEW)

- Essential
- Important
- Don't know/Depends
- Unimportant
- Should not be included

5.8 Self-care for the first aider

Q68. The first aider should have self-care strategies in place to enable them to best take care of themselves while offering support to the person. (NEW)

- Essential
- Important
- Don't know/Depends
- Unimportant
- Should not be included

Section 6: Supporting someone with a history of substance use problems

Section 6: Supporting someone with a history of substance use problems

This section asks you how the first aider should support someone with a prior history of substance use problems.

Please rate how important (from 'essential' to 'should not be included') you think it is that each statement be included in the guidelines.

Please also keep the definitions in mind when rating the statements.

Q69. The first aider should know the difference between a lapse and a relapse. (NEW)

- Essential
- Important

- Don't know/Depends
- Unimportant
- Should not be included

Q70. The first aider should be aware that a person can experience a lapse without it turning into a relapse. (NEW)

- Essential
- Important
- Don't know/Depends
- Unimportant
- Should not be included

Q71. The first aider should be aware that recovery takes time; lapses and relapses can happen and be overcome. (NEW)

- Essential
- Important
- Don't know/Depends
- Unimportant
- Should not be included

Q72. If the person experiences a lapse or relapse, the first aider should not blame or shame them. (NEW)

- Essential
- Important
- Don't know/Depends
- Unimportant
- Should not be included

Q73. The first aider should ask the person if they have an emergency management plan and, if so, follow this. (NEW)

- Essential
- Important

- Don't know/Depends
- Unimportant
- Should not be included

Q74. If the person has previously tried a treatment that didn't work, the first aider should encourage them to consider peer support options. (NEW)

- Essential
- Important
- Don't know/Depends
- Unimportant
- Should not be included

Q75. If the person's basic needs (e.g. food, housing) are not being met, the first aider should direct them to social welfare support. (NEW)

- Essential
- Important
- Don't know/Depends
- Unimportant
- Should not be included

Q76. If the first aider feels they cannot support the person further or needs a break, they should suggest the person uses a helpline for support. (NEW)

- Essential
- Important
- Don't know/Depends
- Unimportant
- Should not be included

Section 7: Professional help

Section 7: Professional help

This section asks you how the first aider should connect the person with professional help.

Please rate how important (from ‘essential’ to ‘should not be included’) you think it is that each statement be included in the guidelines.

Please also keep the definitions in mind when rating the statements.

7.1 Approaching the person about professional help

Q77. The first aider should encourage the person to seek professional help if they are interested in this. (NEW)

- Essential
- Important
- Don't know/Depends
- Unimportant
- Should not be included

Q78. The first aider should discuss with the person the benefits of professional help. (RE-RATE: Item #242)

- Essential
- Important
- Don't know/Depends
- Unimportant
- Should not be included

Q79. If the person is interested but unsure about getting professional help, the first aider should discuss the benefits of professional help with the person. (NEW)

- Essential
- Important
- Don't know/Depends
- Unimportant
- Should not be included

Q80. The first aider should be aware that the sooner professional help commences, the better the person's chances for recovery. (RE-RATE: Item #245)

- Essential
- Important
- Don't know/Depends
- Unimportant
- Should not be included

7.2 Encouraging professional help

Q81. The first aider should ask the person about their preferred sources of help. (NEW)

- Essential
- Important
- Don't know/Depends
- Unimportant
- Should not be included

Q82. The first aider should tell the person that it takes courage to ask for, and to accept, professional help. (RE-RATE: Item #255)

- Essential
- Important
- Don't know/Depends
- Unimportant
- Should not be included

Q83. The first aider should offer to accompany the person to appointments with professionals if appropriate to the relationship. (NEW)

- Essential
- Important
- Don't know/Depends
- Unimportant
- Should not be included

Q84. If the person is experiencing both problems with substance use and mental health, the first aider should support the person to get help for both. (NEW)

- Essential
- Important
- Don't know/Depends
- Unimportant
- Should not be included

Q85. If the person seeks professional help, the first aider should follow up with them to see how the appointment went, if appropriate to the relationship. (NEW)

- Essential
- Important
- Don't know/Depends
- Unimportant
- Should not be included

Q86. If the person is reluctant to seek professional help for their substance use problem, the first aider should encourage them to seek help for any other impacts that substance use is having on their lives, e.g. relationship problems, aggression or financial problems. (RE-RATE: Item #260)

- Essential
- Important
- Don't know/Depends
- Unimportant
- Should not be included

7.3 If the person is unwilling to seek professional help

Q87. If the person does not want professional help, the first aider should respect their decision. (RE-RATE: Item #269)

- Essential
- Important

- Don't know/Depends
- Unimportant
- Should not be included

7.4 Addressing barriers to help seeking

Q88. The first aider should be aware that waiting lists can pose a barrier to accessing professional help, and the person may need to explore alternative treatment options, e.g. online treatment. (NEW)

- Essential
- Important
- Don't know/Depends
- Unimportant
- Should not be included

Q89. The first aider should be aware that cost can pose a barrier to accessing professional help, and they may need help to find affordable options. (NEW)

- Essential
- Important
- Don't know/Depends
- Unimportant
- Should not be included

Q90. The first aider should be aware that the person may have experienced stigma and discrimination and this may be a barrier to seeking further help. (NEW)

- Essential
- Important
- Don't know/Depends
- Unimportant
- Should not be included

Section 8: Interventions

Section 8: Interventions

This section asks you what the first aider should know about interventions.

Please rate how important (from 'essential' to 'should not be included') you think it is that each statement be included in the guidelines.

Please also keep the definitions in mind when rating the statements.

Intervention definition: A group of people coming together to confront a person about their substance use.

Q91. The first aider should not try to carry out an intervention. (NEW)

- Essential
- Important
- Don't know/Depends
- Unimportant
- Should not be included

Q92. The first aider should be aware there is no evidence to suggest that confrontational interventions effectively convince people they have a problem or motivate them to change. (RE-RATE: Item #282)

- Essential
- Important
- Don't know/Depends
- Unimportant
- Should not be included

Section 9: Crisis Situations

Section 9: Crisis situations

This section asks you what the first aider should know in order to recognise a crisis situation and

what to do during a crisis situation.

Please rate how important (from ‘essential’ to ‘should not be included’) you think it is that each statement be included in the guidelines.

Please also keep the **definitions** in mind when rating the statements.

9.1 Recognising a crisis

Q93. The first aider should remove themselves from any crisis situation where they feel unsafe and call for appropriate emergency or crisis services. (NEW)

- Essential
- Important
- Don't know/Depends
- Unimportant
- Should not be included
- I do not have the expertise to answer this question

9.3 Monitor for danger

Q94. If the person is affected by substances, the first aider should try to maintain a safe distance and try to appear non-threatening. (RE-RATE: Item #319)

- Essential
- Important
- Don't know/Depends
- Unimportant
- Should not be included
- I do not have the expertise to answer this question

Q95. If the person is experiencing a crisis, the first aider should discourage the person from taking any more substances. (RE-RATE: Item #321)

- Essential
- Important
- Don't know/Depends

- Unimportant
- Should not be included
- I do not have the expertise to answer this question

Q96. If the person is experiencing a crisis, the first aider should remove any substances from the environment, if safe and practical to do so. (NEW)

- Essential
- Important
- Don't know/Depends
- Unimportant
- Should not be included
- I do not have the expertise to answer this question

9.4 Keep the person safe

Q97. If the person is heavily intoxicated and the first aider feels uncomfortable monitoring them, the first aider should take the person to a sobering up shelter or drug and alcohol service, if available and appropriate to the relationship. (NEW)

- Essential
- Important
- Don't know/Depends
- Unimportant
- Should not be included
- I do not have the expertise to answer this question

Q98. If the first aider has been using substances, they should enlist the help of someone who is sober to assist the person. (RE-RATE: Item #326)

- Essential
- Important
- Don't know/Depends
- Unimportant
- Should not be included
- I do not have the expertise to answer this question

Q99. If the first aider has been using substances, they should try to enlist the help of someone who is sober to assist the person. (NEW)

- Essential
- Important
- Don't know/Depends
- Unimportant
- Should not be included
- I do not have the expertise to answer this question

Q100. The first aider should prevent the intoxicated person from walking off alone, but only if it is safe for the first aider to do so. (RE-RATE: Item #327)

- Essential
- Important
- Don't know/Depends
- Unimportant
- Should not be included
- I do not have the expertise to answer this question

Q101. The first aider should try to prevent the person from taking part in risky or unsafe activities, if safe and practical to do so. (NEW)

- Essential
- Important
- Don't know/Depends
- Unimportant
- Should not be included
- I do not have the expertise to answer this question

Section 9: Crisis situations (continued)

9.7 What to do if the person becomes aggressive

Q102. The first aider should not stay with the person if the first aider's own safety is at risk. (RE-RATE: Item #340)

- Essential
- Important
- Don't know/Depends
- Unimportant
- Should not be included
- I do not have the expertise to answer this question

Q103. If the person becomes aggressive, the first aider should:

	Essential	Important	Don't know/Depends	Unimportant	i
Move slowly. (RE-RATE: Item #362)	○	○	○	○	
Try to ensure there is a physical barrier (e.g. a desk) between themselves and the person. (NEW)	○	○	○	○	
Try to maintain open body language. (NEW)	○	○	○	○	
Try to use other possible de-escalation methods before calling the police. (NEW)	○	○	○	○	

Q104. The first aider should only call the police if all other avenues of de-escalation have been exhausted and the person is at risk of harming themselves or others. (RE-RATE: Item #370)

- Essential
- Important
- Don't know/Depends
- Unimportant
- Should not be included
- I do not have the expertise to answer this question

Q105. If the person becomes aggressive and the first aider needs to call the police, the first aider should tell the person they are doing so. (NEW)

- Essential
- Important
- Don't know/Depends
- Unimportant
- Should not be included
- I do not have the expertise to answer this question

9.8 Adverse psychological reactions

Q106. The first aider should be able to recognise adverse psychological reactions to substances. (RE-RATE: Item #374)

- Essential
- Important
- Don't know/Depends
- Unimportant
- Should not be included
- I do not have the expertise to answer this question

Section 10: Adolescents

Section 10: Adolescents

This section asks you what the first aider should consider if an adolescent is experiencing substance use problems.

Please rate how important (from ‘essential’ to ‘should not be included’) you think it is that each statement be included in the guidelines.

Please also keep the definitions in mind when rating the statements.

Q107. The first aider should discuss with the adolescent how substance use can lead to substance use problems. (NEW)

- Essential
- Important
- Don't know/Depends
- Unimportant
- Should not be included

Q108. The first aider should discuss the health risks and potential consequences of using substances with the adolescent. (NEW)

- Essential
- Important
- Don't know/Depends
- Unimportant
- Should not be included

Q109. If the first aider is a legal guardian and suspects the adolescent is experiencing problem substance use, they should consider searching through their things to find evidence of substance use. (NEW)

- Essential
- Important
- Don't know/Depends
- Unimportant
- Should not be included

Powered by Qualtrics

Round 3 Survey

R3 Instructions

Round 3 survey: Updating the Mental Health First Aid guidelines for problem substance use

Your participation

Thank you for your participation in this project so far.

As advised, participation in this project involves completing three rounds of online surveys. Thank you for recently completing the second survey. It is now time to complete the third and final survey.

Purpose of the research

The aim of this current research project is to update the mental health first aid guidelines for how a member of the public should give assistance to a person who is experiencing problem alcohol or other drug use.

Instructions

Your task is to complete the survey by rating each statement according to how important you believe it is for inclusion in the guidelines for providing mental health first aid for someone who is experiencing problem alcohol or other drug use. This involves re-rating some items from Round 2.

Please keep in mind that the guidelines will be used by the general public. The statements need to be rated according to their importance for someone WITHOUT a counselling or clinical background.

This questionnaire should take approximately 10 - 15 minutes to complete. You can complete the survey in two or more sittings. Your answers are saved when you click 'Next' at the bottom of the page. This marks your page and you can begin again at a later date on the next page. Please be aware that once you have logged on and started responding you must complete the questionnaire on the same computer.

How this questionnaire was developed

The following questionnaire is comprised of statements that appeared for the first time in the Round 2 survey. These statements are being re-rated because they were neither endorsed nor rejected by the expert groups. A statement is rerated when 70% - 79% of panel members in one or more expert groups rated it as essential or important, and 80% or more of panel members from the remaining expert group(s) rated it as essential or important.

As advised, you may wish to use information from your Round 2 Report to inform how you respond to re-rate statements in this round. Please note, the statement numbers (e.g., Statement #1) and order in which they are presented in the Round 2 Report correspond to those in the Round 3 survey.

Consent to participate

It is important for you to know that participation in this study is completely voluntary. You are not under any obligation to participate and can withdraw at any time.

We would like to thank you for your time and effort, and encourage you to provide us with feedback on this process.

Who can I contact if I have any concerns about the project?

This research project has been approved by the Human Research Ethics Committee of The University of Melbourne. If you have any concerns or complaints about the conduct of this research project which you do not wish to discuss with the research team, you should contact the Manager, Human Research Ethics, Research Ethics and Integrity, University of Melbourne, VIC 3010 on Tel: +61 3 8344 2073 or Email: HumanEthics-complaints@unimelb.edu.au. All complaints will be treated confidentially. In any correspondence, please provide the ethics ID number. **The ethics ID number of the research project is 1851765.**

For more information

You received a Plain Language Statement when you expressed interest in this project (also available here). Please refer to this for more details about this study. You may also contact Judith Wright via email for further information: wright.j@unimelb.edu.au.

Definitions

Definitions used in this survey

Mental health first aid is the help provided to a person who is developing a mental health problem, experiencing a worsening of an existing mental health problem or in a mental health crisis. The first aid is given until appropriate professional help is received or the crisis resolves.

The person: someone who is experiencing substance use problems.

The first aider: a concerned family member, friend, work colleague or community member, who provides help to a person experiencing substance use problems.

Professional: a broad range of relevantly trained health professionals. This could include a mental health professional, GP/family doctor, hospital emergency staff, ambulance officer or

paramedic.

Emergency services: services that respond to and deal with emergencies when they occur, e.g. emergency medical services (ambulance) or law enforcement (the police).

Substances: psychoactive compounds, including alcohol or other drugs. Substances may be legal or illegal.

Substance use problems/Problem substance use: a pattern of harmful use of substances that has the potential to negatively impact a person's physical and mental health, relationships, employment, finances, and the safety of themselves and others. They may or may not have a substance use disorder.

Substance dependence: refers to heavy, regular substance use that results in the person having difficulties controlling the amount they use and experiencing symptoms (such as anxiety) when they stop using or use less than usual. Substance dependence occurs when someone feels they need to use substances in order to get through their day or week.

Lapse: a short (or temporary) departure from a person's substance use goals followed by a return to their goals.

Relapse: a return to previous levels of substance use after a period of sustained reduction or avoidance of substances.

Withdrawal: refers to a physical condition that occurs when a person, who has been using substances heavily or for extended periods of time, stops using or uses substantially less than usual. Symptoms of withdrawal include severe anxiety, headaches and tremors or shakes. Withdrawal can be a life-threatening condition.

Overdose: is when too much of a substance/s is taken causing harmful effects on the body. Overdoses may be accidental or intentional and can involve substances that are prescription, over-the-counter, legal, or illegal. A person can overdose on many substances, including alcohol (alcohol poisoning), stimulants, pain medication or a mix of substances. An overdose can lead to serious medical complications, including death.

Overview of the questionnaire

This questionnaire contains 14 statements across the following sections:

Section 2: When does the person need help? (4 statements)
Section 3: Approaching the person (2 statements)

Section 4: Providing information (1 statement)
Section 5: Providing support (1 statement)
Section 7: Professional help (2 statements)
Section 8: Interventions (1 statement)
Section 9: Crisis situations (3 statements)

Information about you

Information about you

Q1. What is your name?

(This allows us to determine who has completed the Round 2 survey and is therefore eligible to participate in Round 3. Your name will be deleted from your data when the project is complete).

Section 2: When does the person need help?

Section 2: When does the person need help?

This section contains **4 statements** about what the first aider should know in order to recognise when the person needs help.

Please rate how important (from 'essential' to 'should not be included') you think it is that each statement be included in the guidelines.

Please also keep the definitions in mind when rating the statements

2.2 Prioritising others' safety

Statement #1: If the first aider becomes aware that the person's substance use is placing the safety of other adults (e.g. partner or adult family members) at risk, the first aider should offer them information on resources available to them.

- ○ Essential
- ○ Important
- ○ Don't know/Depends

- ○ Unimportant
- ○ Should not be included

2.3 Warning signs

The first aider should know the following warning signs for recognising substance use problems:

	Essential	Important	Do know/D
**Statement #2:** The person behaves in uncharacteristic ways.	○	○	○
**Statement #3:** The person spends increasingly less time with family and friends.	○	○	○
**Statement #4:** The person is not meeting home, social or work obligations and responsibilities.	○	○	○

Section 3: Approaching the person

Section 3: Approaching the person

This section contains **2 statements** about what the first aider should know and do when approaching the person.

Please rate how important (from ‘essential’ to ‘should not be included’) you think it is that each statement be included in the guidelines.

Please also keep the definitions in mind when rating the statements

3.9 What to do if the person does not believe they have a problem

Statement #5: If the person does not believe they have a problem, the first aider should continue to provide social support.

- ○ Essential
- ○ Important
- ○ Don't know/Depends
- ○ Unimportant
- ○ Should not be included

Statement #6: If the person does not believe they have a problem, the first aider should encourage the person to reduce harms related to their substance use.

- Essential
- Important
- Don't know/Depends
- Unimportant
- Should not be included

Section 4: Providing Information

Section 4: Providing information

This section contains **1 statement** about what information the first aider should provide to the person.

Please rate how important (from 'essential' to 'should not be included') you think it is that this statement be included in the guidelines.

Please also keep the definitions in mind when rating the statements.

4.2 Harm reduction

Statement #7: The first aider should offer the person information about harm reduction strategies.

- Essential
- Important
- Don't know/Depends
- Unimportant
- Should not be included

Section 5: Providing support

Section 5: Supporting the person

This section contains **1 statement** about how the first aider should support the person.

Please rate how important (from 'essential' to 'should not be included') you think it is that this statement be included in the guidelines.

Please also keep the definitions in mind when rating the statements.

5.4 Managing social pressure

Statement #8: The first aider should tell the person that the people who care about their well-being will accept their decision not to use.

- ◯ Essential
- ◯ Important
- ◯ Don't know/Depends
- ◯ Unimportant
- ◯ Should not be included

Section 7: Professional help

Section 7: Professional help

This section contains **2 statements** about how the first aider should connect the person with professional help.

Please rate how important (from 'essential' to 'should not be included') you think it is that each statement be included in the guidelines.

Please also keep the definitions in mind when rating the statements.

7.2 Encouraging professional help

Statement #9: If the person is experiencing both problems with substance use and mental health, the first aider should support the person to get help for both.

- ◯ Essential

- Important
- Don't know/Depends
- Unimportant
- Should not be included

Statement #10: If the person seeks professional help, the first aider should follow up with them to see how the appointment went, if appropriate to the relationship.

- Essential
- Important
- Don't know/Depends
- Unimportant
- Should not be included

Section 8: Interventions

Section 8: Interventions

This section contains **1 statement** about what the first aider should know about interventions.

Please rate how important (from 'essential' to 'should not be included') you think it is that this statement be included in the guidelines.

Please also keep the definitions in mind when rating the statements.

Intervention definition: A group of people coming together to confront a person about their substance use.

Statement #11: The first aider should not try to carry out an intervention.

- Essential
- Important
- Don't know/Depends
- Unimportant
- Should not be included

Section 9: Crisis situations

This section contains **3 statements** about what the first aider should know in order to recognise a crisis situation and what to do during a crisis situation.

Please rate how important (from ‘essential’ to ‘should not be included’) you think it is that each statement be included in the guidelines.

Please also keep the **definitions** in mind when rating the statements.

9.4 Keep the person safe

Statement #12: If the first aider has been using substances, they should try to enlist the help of someone who is sober to assist the person.

- Essential
- Important
- Don't know/Depends
- Unimportant
- Should not be included
- I do not have the expertise to answer this question

Statement #13: The first aider should try to prevent the person from taking part in risky or unsafe activities, if safe and practical to do so.

- Essential
- Important
- Don't know/Depends
- Unimportant
- Should not be included
- I do not have the expertise to answer this question

9.7 What to do if the person becomes aggressive

Statement #14: If the person becomes aggressive, the first aider should try to use other possible de-escalation methods before calling the police.

- ○ Essential
- ○ Important
- ○ Don't know/Depends
- ○ Unimportant
- ○ Should not be included
- ○ I do not have the expertise to answer this question

Powered by Qualtrics
