# Supplementary material for: Redevelopment of mental health first aid guidelines for substance use problems: a Delphi study
Source: BMC Psychol. 2024 Feb 13;12:70. doi: 10.1186/s40359-024-01561-8 (PMC10865545; doi:10.1186/s40359-024-01561-8)
Supplement: Supplementary file 3 — Additional File 3: Substance use problems: MHFA guidelines [file 40359_2024_1561_MOESM3_ESM.docx]

# **What are substance use problems?**

A substance use problem refers to a pattern of harmful use of substances (e.g. alcohol, cannabis, ecstasy, amphetamines, opiates, cocaine and/or heroin) that has the potential to negatively impact a person's physical and mental health, relationships, employment, finances, and the safety of themselves and others. A substance use problem is not just a matter of how much of a substance a person uses, but how their use affects their life and the lives of those around them. Do not assume that any use of substance(s) means that a person has a substance use problem, e.g. being dependent.

There are both social and individual risk factors for substance use problems. These include, for example, using substances at an early age, having a family history of substance use problems, exposure to a highly addictive substance, and peer pressure. Traumatic experiences may have also contributed to the person’s substance use problems.

There is a high co-occurrence between substance use problems and other mental health problems, e.g. many people who develop substance use problems may also experience depression or post-traumatic stress disorder. Substances are often used to cope with underlying emotional distress or mental illness. This is called ‘self-medication’. Mental health problems can be caused or made worse by substance use. If the person has underlying emotional distress or mental health problems, these may need to be addressed for them to reduce or stop their substance use.

| **POTENTIAL CONSEQUENCES OF SUBSTANCE USE PROBLEMS** |
| --- |
| There are short-term and long-term consequences of substance use problems. These can include physical, mental or social problems. For example:   - Adverse effects on judgement and decision-making - Family or social difficulties, e.g. relationship, financial problems - Difficulties with educational or work performance - Legal problems - Injuries while using substances, e.g. from accidents, falls, violence, road trauma - Physical health problems, e.g. liver disease, brain damage, cancer - Mental health problems, e.g. panic attacks, psychosis, suicidal thoughts and behaviours - Difficulty controlling the amount of time spent using or the quantity used - Needing more of the drug to get the same effect - Problems in cutting down or controlling use - Experiencing unpleasant symptoms when stopping or reducing use. |

Different substances can affect a person’s behaviour differently. For example:

- Alcohol reduces a person’s inhibitions and may lead them to behave in a way they normally would not.
- Depressant substances, such as alcohol and prescription medications with sedating properties, reduce a person’s arousal and stimulation, making them appear relaxed and drowsy.
- Stimulants, such as methamphetamine and cocaine, speed up the messages travelling between the brain and body, making the person appear more awake, alert, confident or energetic than usual.

**WHEN DOES THE PERSON NEED HELP?**

It is important for you to be able to recognise the signs of substance use problems. These include the person:

- Needing to use substances to help deal with certain situations.
- Acknowledging they think a lot about substance use and when they’ll next get a chance to use.
- Appearing to prioritise acquiring and using substances over other parts of their life, e.g. work and social life.
- Not meeting home, social or work obligations and responsibilities.
- Becoming isolated from both family and friends.
- Engaging in criminal conduct, fighting or driving under the influence of the substance(s).
- Using increasingly larger amounts of substances and using more often over time.
- Continuing their substance use despite it causing distress to themselves and the people around them.
- Being unable to stop or reduce their substance use even if they say they want to.
- Spending a lot of time looking for a substance, using it and recovering from using it.
- Using the substance(s) in risky situations, e.g. driving.
- Using the substance(s) alone, in the morning or for hours on end.
- Using the substance(s) to numb negative feelings or cope with mental health problems.

You should also know common physical signs and symptoms of substance use problems.

Use of prescription medication can sometimes become a problem. Signs that this is occurring are^1^:

- Taking more medication than prescribed or directed on the packet, either in one dose or over time.
- Taking medication in a non-recommended way, such as injecting or snorting.
- Using medication without a prescription and ongoing medical supervision.
- Combining prescription medication with other drugs, e.g. alcohol.
- Undertaking activities that medication affects, like driving, working or looking after children.
- Sharing prescription medication with friends, family or colleagues.

*^1^ Adapted from: Alcohol and Drug Foundation. Reducing Risk: Pharmaceuticals.* [*https://adf.org.au/reducing-risk/pharmaceuticals/*](https://adf.org.au/reducing-risk/pharmaceuticals/)

# Plan the approach

There are a wide range of reasons why people use substances (e.g. to relax or for enjoyment) and develop substance use problems, e.g. stopping use leads to unpleasant effects. Before you approach the person, reflect on their situation, organise your own thoughts, and decide what you want to say to them. Have some helpful contact numbers with you so the person can call for confidential help or more information, if they are willing to receive it.

If you need support to provide first aid you can:

- Contact health professionals who specialise in substance use problems for information and support to determine how best to approach the person about your concerns and provide assistance.
- Consult with others who have dealt with substance use problems about effective ways to help.
- Contact a helpline for support in assisting someone with substance use problems.

**Prioritise self-care**

Your own self-care is as important as the care offered to the person. You should have self-care strategies in place to enable you to take care of yourself while offering support. Use these self-care strategies if you feel upset, overwhelmed, stressed or unwell after helping the person. Alcohol and drug helplines are often an available support option for supporters of people with substance use problems who feel stigmatised or prefer to remain anonymous. You should also access informal and formal help sources for yourself, as you will be more likely to maintain your support-giving role.

**Prioritise the safety of others impacted by the person's substance use**

If there are any children being affected by the person’s substance use, you should make the children’s safety and well-being a priority. If you become aware that the person’s substance use is placing the safety of others (e.g. partner or family members) at risk, you should:

- Prioritise keeping these people safe, if practical and safe to do so.
- Offer information on resources available to them.

**Consider potential outcomes of approaching the person**

Consider that the person might not believe, or might deny, that they have a substance use problem. Do not expect them to change right away; this conversation may be the first time they have thought of their substance use as a problem.

Know that substance dependence is a mental health problem and not a wilful choice. Be aware that the person may be very sensitive to stereotyping and the judgements of others. They may be reluctant to admit they have a problem for a number of reasons, e.g. due to stigma, fear of being labelled, and effects on relationships.

The person may find it hard to disclose or discuss their substance use because they may feel embarrassed or ashamed. They may also not recall events that occurred while they were intoxicated, i.e. they may have blacked out. Do not expect them to tell you everything about their substance use.

The person may resist your help for a number of reasons. For example, they might feel that you are trying to take away their “right to drink”. They may also not want to reduce or stop their substance use.

They might react aggressively when approached about their substance use, give excuses, get angry, or try to blame you or other family members for their substance use problem. Try not to take personally things the person says or does when they are affected by substances.

# **Making the approach**

If you are concerned about someone’s substance use, choose an appropriate time and setting to approach them, e.g. somewhere quiet and private at a time when there will be no interruptions. Remember that you may need to have several conversations with them.

Ultimately, the person is the only one who can make the decision to change their substance use behaviour. You cannot force them to accept help if they are not ready to do so. A person’s willpower is not always enough to help them overcome substance use problems.

Understand that the person may not know why they use substances and the environment in which they use can make it harder or easier for them to change their substance use behaviour. Also, substances can interfere with normal thought processes making it difficult to understand that change is necessary.

It is possible for a person to change their substance use. Remember, any positive change towards reducing substance use is beneficial. Changing habits associated with substance use problems is a process that typically takes time; it is not easy and requires repeated efforts. In trying to reduce or change their substance use, a person may experience emotional and physical stress.

**How to communicate with the person**

- Talk to them using non-judgmental communication in a calm, reasonable and non-confrontational manner that is assertive but not aggressive.
- Use “I” statements instead of “you” statements, e.g. “I feel worried/angry/frustrated when you...” instead of “You make me feel...", and stick to the point without getting drawn into arguments about other issues.
- Let the person know that you will listen without judging them and will try to understand their perception of their substance use.
- Ask them whether they consider their substance use a problem and about areas of their life the substance use may be affecting, e.g. their mood, work performance and relationships.
- Focus the conversation on the person's behaviour rather than their character, e.g. “Your drinking seems to be getting in the way of your friendships” rather than “You're a useless drunk".

Remember, giving advice alone may not help the person change their substance use behaviour. Be aware that they may already know a lot about substance use problems. Also, it is important that you have some knowledge about cultural norms around substance use for the person you are assisting.

If the person does not believe they have a problem, let them know that you are available to talk in the future and encourage them to reduce harms related to their substance use.

### **How to support the person if they are willing to change**

Ask the person if they want help to change their substance use behaviour. If they do, ask them what type of help and support they would find most helpful. Offer to help them and discuss what assistance and support you are willing to provide. Establish boundaries of acceptable and unacceptable behaviour with them.

Communicate a sense of hope and belief to the person that they can change their substance use problems. Tell them they are capable of change if that is what they want. Be positive and encouraging of any efforts they make to change their substance use problems. Acknowledge the positive things they do and achieve. Help the person to stay focused on positive goals that do not involve substances. Encourage the person to keep trying if they have a setback.

Reassure them that they are not alone and many people have problems with substances. If they have previously tried to make a change to their substance use, discuss with them what was helpful and what wasn’t.

# **Providing information and support**

Offer to help the person get information on a range of local treatment and support options. Allow them to decide which would be most appropriate or useful for them. Be prepared to offer information for group support or self-help strategies if the person needs them. Make sure any information you provide is from a reputable source. Let them know they can contact a helpline for support when they are ready for change.

If they are open to receiving information, you should:

- Offer them some information about substance use problems.
- Provide some basic facts about substance use problems, e.g. how common they are, the associated risks, available treatments.
- Discuss with them some risks associated with substance use problems.
- Help them get some information on how to change their substance use problems.

### **Harm reduction**

Offer the person information about harm reduction strategies. Harm reduction approaches aim to prevent or reduce the negative consequences associated with substance use and reduce other related risk factors. For example:

- Needle and syringe programs protect people who inject drugs from HIV and other blood-borne viruses that are transmitted via used needles and syringes by making sterile equipment accessible.
- Methadone and buprenorphine programs are effective in helping people reduce their dependence on heroin and pharmaceutical opioids. They can reduce substance-related harms such as illicit opioid drug use, injecting and substance related illness and mortality.

There are some ways of using substances that are less harmful than other ways. These include, for example, controlled drinking, pill testing, not injecting and not sharing needles or equipment.

If the person is interested in harm reduction strategies, ask if you can assist with this in any way. Encourage them to avoid engaging in activities that may pose a risk to others while using substances, e.g. driving, working, or looking after children.

If the person is injecting drugs, tell them how they can find out about harm reduction programs, e.g. needle and syringe programs. Tell them that it is never okay to share injecting equipment (i.e., needles, syringes, tourniquets, filters, spoons, waters) with someone else, even if they say “they’re safe” or “they’re clean”, are family, or if the person knows them well.

| **IF THE PERSON IS PREGNANT, BREASTFEEDING OR SPENDING TIME WITH CHILDREN** |
| --- |
| If the person is pregnant or breastfeeding, discuss that using substances during pregnancy is unsafe for the baby and encourage them to seek appropriate professional help as soon as possible. If the person is pregnant and wants to stop or reduce their substance use, tell them that medical help is important to do this safely, as suddenly stopping substance use without medical supervision can be dangerous for the unborn baby. Encourage the person not to use substances around children. |

**What to avoid**

Forcing a person to admit they have a substance use problem may cause conflict. When talking with the person:

- Do not lecture them.
- Do not apply any stigmatising labels to them such as 'addict’, ‘alcoholic’ or ‘drunk’.
- Do not press them to talk if they don't want to talk.
- Do not force them to admit they have a substance use problem.
- Do not bribe them to change their substance use.
- Do not criticise the person’s substance use behaviour.
- Do not use scare tactics.
- Do not demonise substances or give exaggerated messages about substance use.
- Do not make them feel guilty or ashamed because of their substance use.
- Do not say unrealistic things, e.g. "Just don't drink", or “You'll feel better really soon”.
- Do not minimise their substance use problems, e.g. “Everyone drinks", or “You don't look like an addict.”
- Do not give glib reassurances, e.g. “Everything happens for a reason" or “Time heals all wounds.”

# **Encourage other supports**

Be aware that even doing small things to help can make a difference to the person’s substance use. Also, if you maintain a good relationship with them, you may have a beneficial effect on their substance use behaviour. Encourage the person to reach out and spend time with friends and family who support their efforts to change their substance use and don’t use substances. Also, encourage them to talk to someone they trust about their problems, not just their substance use, e.g. they might talk to a friend, family member or community support worker.

Detoxification is only part of recovery; lifestyle changes are required to alter substance use behaviours. Discuss with the person what realistic lifestyle changes they could adopt without overwhelming them with options, and encourage them to find ways of coping when they feel the urge to use substances, e.g. talking to someone they trust. Be supportive of the positive choices the person makes, such as playing sport or spending time with people that don’t use substances. Reassure the person that they do not need to explain to others why they are not using substances and recommend they have an “exit plan” to use in social situations where they are tempted to use substances.

Encourage the person to do more of what keeps them feeling well (e.g. connecting with friends and family) and find healthy ways to feel good instead of using substances. Also encourage the person to try and get a good amount of sleep so their body can work to repair itself while they are trying to change their substance use problem.

If there is a reason you cannot assist the person effectively, help them find someone else who can.

| **WHEN TO DISCLOSE** |
| --- |
| You should not disclose the person's substance use problem to a friend, family member, or professional unless:   - The person has given you consent. - The person is at risk of harming themselves or others. - There is a child or young person being placed at risk because of the person's substance use problem. |

# **Supporting the person through relapse**

Know that recovery takes time; lapses and relapses can sometimes happen and be overcome. A lapse is a short return to substance use, and a relapse is a return to substance use which a person previously managed or quit completely. A person can experience a lapse without it turning into a relapse. Let the person know they can contact a helpline for support if they experience distress or a craving.

The person may relapse once or several times before changing their substance use patterns. If they do experience a lapse or relapse, do not blame or shame them. Ask the person if they have an emergency management plan, if so, follow this.

You should know the stages of change and the process it takes for somebody to change their behaviour.

| **STAGES OF CHANGE** |
| --- |
| A person who has a substance problem may not be ready to change. Major behaviour changes take time to be achieved and often involve the person going through a number of stages. There are six "stages of change", and the person may move back and forth between the stages at different times.    **Stage 1: Pre-contemplation**  The person does not consider their substance use to be a problem.    **Stage 2: Contemplation**  The person thinks about changing, cutting down, or stopping their substance use.    **Stage 3: Preparation**  The person decides to make a change and makes plans for carrying out changes, e.g. decides exactly which changes to make, puts supports in place.  **Stage 4: Action**  The person makes changes.    **Stage 5: Maintenance**  The person keeps up the changes they began in the action stage.  **Stage 6: Termination**  The person is certain they will not return to their previous use of substances and has no desire to do so.  Note: Relapse can occur during the ‘Action’ and ‘Maintenance’ stages in which the person returns to an earlier stage, e.g. ‘Contemplation’.   - *Source: Prochaska JO, Velicer WF. The transtheoretical model of health behavior change. American journal of health promotion. 1997 Sep;12(1):38-48.* |

If the person has previously tried a treatment that didn't work, encourage them to try seeking professional help again because new options may be available. Be aware that it may take some time to find a health professional with whom the person is able to establish a good relationship. If this is the case, encourage the person not to give up. Direct the person to social welfare support if their basic needs (e.g. food, housing) are not being met.

# **If the person does not want to change**

Be patient while waiting for the person to accept they have a problem. Tell them what behaviour you are willing to accept from them, for example, explain that they can come to your home for a social visit, but only when they are not intoxicated. Assure them that you will be there if they want help or to talk again in the future.

If the person does not want to change their substance use:

- Do not try to control them by bribing, nagging, threatening or crying.
- Do not use negative approaches that are unlikely to promote change, e.g. lecturing the person or making them feel guilty.
- Do not take on their responsibilities, except if not doing so would cause harm, e.g. to their own or other’s lives.
- Do not deny their basic needs.
- Do not cover up or make excuses for them.
- Do not use substances with them.
- Do not take responsibility for their recovery journey.
- Try not to feel guilty or responsible for their decision to keeping using substances.

# **Professional help**

There are effective interventions for substance use problems. Treatment options and support services include education, counselling, therapy, rehabilitation and self-help groups. Some treatment programs emphasise a substance-free lifestyle, while other programs aim to reduce the person's substance use. Be aware of available treatment options in the person’s local area.

Ceasing to use a substance without medical advice can be dangerous. If the person has been drinking or using heavily for a long period and decides to stop suddenly, encourage them to seek medical attention as this can be very dangerous to their health. **If they have been drinking for a long period, tell them not to suddenly stop drinking without seeking medical help to avoid the possibility of withdrawal, which can be fatal.**

**Encouraging professional help**

Ask the person if they would like to get professional help and encourage them to seek it if they are interested in this. Discuss the benefits of professional help with them, even if the person is interested but unsure about getting professional help.

Explain to the person that there are several treatment approaches available. Ask them about their preferred sources of help and do not pressure them into a single type of treatment. Give the person information about local options for professional help. Be prepared for them to respond negatively when you suggest professional help. Don’t use a confrontational or coercive approach when trying to get them to seek professional help.

If the person is experiencing any symptoms of mental illness, encourage them to seek professional help, whether the symptoms are substance-use related or not. If they are experiencing problems with both substance use and mental health, support them to get help for both.

Encourage the person to call emergency services if they have an adverse reaction while using substances.

**If the person is unwilling to seek professional help**

Keep in mind that it is common for people with substance use problems to resist seeking professional help. Professional help cannot be forced upon a person except under certain circumstances, e.g. if the police are called or following a medical emergency.

If the person is unwilling to seek professional help, do not use negative approaches to get the person to do this, such as threats, accusations, nagging or ridiculing. Instead:

- Be prepared to talk to the person again in the future.
- Be patient and remain optimistic; there will be opportunities to suggest professional help again in future.
- Continue to suggest professional help to the person if they are putting themselves or others at risk of harm.

If the person does not seek professional help, you should not feel like you have failed. It is ultimately the person's decision to get professional help and they may only accept professional help when the consequences of their substance use become serious enough. Give the person a card or the phone number of a service they can use when they feel ready.

**Addressing barriers to support professional help-seeking**

Barriers to help seeking and treatment that may prevent the person from seeking professional help include waiting lists, cost, risk to privacy and confidentiality, shame and previous experiences of stigma and discrimination. You can help to overcome these barriers by:

- Reassuring the person that professional help is confidential.
- Telling the person that there is no shame in getting help to change their substance use problems.
- Explaining that the goal of professional help may be to help the person to find ways to reduce their substance use, rather than to make the person quit altogether.
- Explaining to the person that they do not need to be an ‘alcoholic’ or ‘addict’ to benefit from talking to a professional.

Be compassionate and patient while waiting for the person to accept they need help.

# **Interventions**

An intervention is when a group of people come together to confront a person about their substance use. There is no evidence to suggest that interventions effectively convince people they have a problem or motivate them to change. An intervention may not be successful and the person may respond negatively to the intervention.

# **Substance affected states and crisis situations**

It is important for you to be able to recognise the signs of intoxication. It is often difficult to differentiate between the effects of different substances on behaviour. The effects of substances vary from person to person depending on factors such as speed of consumption, whether they have eaten anything, body size and age. Symptoms of other medical conditions can also mimic the effects of substance use. Illicit substances can have unpredictable effects as they are not manufactured in a controlled way.

Be aware that only time will reverse the effects of intoxication. It is a myth that consuming caffeine, sleep, walking around or having a shower will make the effects pass more quickly.

It is also important for you to be able to recognise the signs of withdrawal. These include insomnia, irritability, changing moods, depression, anxiety, aches and pains, cravings, fatigue, hallucinations, nausea and seizures. The person may be hot and cold, have goosebumps, or have a runny nose as if they have a cold.

## **What to do if the person is affected by substances**

### *Stay calm and communicate effectively*

Talk with the person in a calm, reassuring and respectful manner. Use simple, clear language and repeat yourself when necessary. Use positive words (e.g. ‘I'm here with you’) instead of negative words (e.g. ‘don't fight’) which they may overreact to. Do not speak in an angry manner or attempt to engage the person in a serious conversation about their substance use, nor laugh at, make fun of, or provoke them while they are intoxicated.

### *Monitor the person*

Assess the situation for potential danger and ensure the person, yourself and others are safe. Try to maintain a safe distance and appear non-threatening. Monitor them and their environment to prevent injury. Ask them if they have taken any medications or other drugs, in case their condition deteriorates into a medical emergency. Also, be aware that substance use can mask pain from injuries. Watch them for signs of increasing aggression.

### *Keep the person safe*

Stay with the person and ensure they are not left alone. Be aware that when intoxicated they may engage in a wide range of risky activities, such as having unprotected sex, arguments or fights, or driving a car. They are also at risk of being a victim of crime. You should organise a safe mode of transport for them.

If the intoxicated person needs to be contained, sobering up shelters and drug and alcohol resource centres are preferable to police lock-ups. This is because they can help the person stay safe, learn about their substance use and its risks, and get some professional help.

**If the person is having an adverse psychological reaction**

Mental health problems can be caused or exacerbated by substance use. It can be difficult to differentiate between the symptoms of mental illness and substance-affected behaviour.

*Panic attacks*

If the affected person is anxious and panicky, take them to a quiet environment away from crowds, loud noise and bright lights. Refer to the Mental Health First Aid Guidelines for how to assist someone experiencing a panic attack, [see here](https://mhfa.com.au/mental-health-first-aid-guidelines).

*Psychosis or psychotic symptoms*

It is important to be aware that any psychotic symptoms could be substance related. Refer to the Mental Health First Aid Guidelines for how to assist someone experiencing psychosis or psychotic symptoms, [see here](https://mhfa.com.au/mental-health-first-aid-guidelines).

*Suicidal thoughts or behaviours*

An intoxicated person may be at a higher risk of attempting suicide. You should watch for the warning signs for suicide. Refer to the Mental Health First Aid Guidelines for how to assist someone experiencing suicidal thoughts or behaviours, [see here](https://mhfa.com.au/mental-health-first-aid-guidelines).

**Medical emergencies**

It is important for you to be able to recognise signs of a substance-related medical emergency, e.g. alcohol poisoning, overdose and severe withdrawal. Be aware that poisoning and overdose depend on the type and quantity of substance used, and this is different for every person.

You should seek appropriate emergency help in the following situations:

- The physical condition of the person using substances is getting worse, e.g. they become unconscious or start vomiting uncontrollably.
- The person displays symptoms of severe withdrawal such as paranoia, confusion, tremors, disorientation or seizures.
- You have removed yourself from the situation because you feel unsafe.

It is important that you know your local emergency services physical first aid guidelines for intoxication and overdose.

**What to do if the person is aggressive**

If the person becomes aggressive, assess the risks to yourself, the person and others before trying to assist. Do not stay with the person if your own safety is at risk.

Prioritise your own safety by keeping a safe distance from the person or, if you are inside, try to keep the exits clear so they do not feel penned in and you and others can get away easily if needed.

Try to remain as calm as possible and de-escalate the situation with the following techniques:

- Talk in a calm, non-confrontational manner and use open body language.
- Speak slowly and confidently, and repeat things if necessary using a gentle, caring tone of voice.
- Try to provide the person with a quiet environment away from noise and other distractions and make sure the area is as safe as possible, e.g. by removing dangerous objects.
- Try and use the person's name, e.g. "Jason, can you tell me what is going on for you?"
- Reassure the person that you are there to help.
- Acknowledge the person's agitation, e.g. "I can see that you are upset."
- Ask the person what they want and then repeat what they requested.
- Tell the person what you are going to do before doing it, e.g. calling for help or asking others to leave.
- Consider taking a break from the conversation to allow the person a chance to calm down.
- Try not to provoke the person. Avoid asking too many questions or arguing with them.
- Avoid displaying nervous behaviour, e.g. shuffling your feet, fidgeting or making abrupt movements.

Continually reassess whether what you are doing is helping; if not, then you should try something different. Get help if the person is going to hurt themselves or someone else. If violence occurs, seek appropriate emergency assistance. Only call the police if all other avenues of de-escalation have been exhausted and the person is at risk of harming themselves or others.

# **Additional considerations for supporting an adolescent**

Adolescence is a critical period of development which can be adversely affected by the use of substances. For example, using substances can damage the brain and result in an inability to experience pleasure or reward, difficulties with controlling impulses, and memory and learning problems. Discuss with the adolescent how substance use can lead to substance use problems and related health risks, and the potential consequences of using substances.

**PURPOSE OF THESE GUIDELINES**

These guidelines are designed to help members of the public to provide first aid to someone who is experiencing substance use problems. The role of the first aider is to assist the person until appropriate professional help is received or the crisis resolves.

**DEVELOPMENT OF THESE GUIDELINES**

These guidelines are based on the expert opinions of people with lived experience of

substance use problems (consumers and carers) and mental health professionals (clinicians, researchers, and educators) who are from Australia, Canada, Finland, the Netherlands, New Zealand, and the United Kingdom.

**HOW TO USE THESE GUIDELINES**

It is important to tailor your support to the needs of the person you are helping. These guidelines are a general set of recommendations only, and most suitable for providing mental health first aid in high-income countries with developed health systems.

These guidelines have been developed as part of a suite of guidelines about how to best assist a person with mental health problems. These other guidelines can be downloaded from:

[mhfa.com.au/resources/mental-health-first-aid-guidelines](http://mhfa.com.au/resources/mental-health-first-aid-guidelines)

Although these guidelines are copyright, they can be freely reproduced for non-profit purposes provided the source is acknowledged. Please cite these guidelines as follows:

Mental Health First Aid Australia. Substance Use Problems: MHFA Guidelines. Melbourne: Mental Health First Aid Australia; 2023.

Enquiries should be sent to: mhfa@mhfa.com.au
